# Molecular pathogenesis of Spondylocheirodysplastic Ehlers-Danlos syndrome caused by mutant ZIP13 proteins

**DOI:** 10.15252/emmm.201303809

**Published:** 2014-07-09

**Authors:** Bum-Ho Bin, Shintaro Hojyo, Toshiaki Hosaka, Jinhyuk Bhin, Hiroki Kano, Tomohiro Miyai, Mariko Ikeda, Tomomi Kimura-Someya, Mikako Shirouzu, Eun-Gyung Cho, Kazuhisa Fukue, Taiho Kambe, Wakana Ohashi, Kyu-Han Kim, Juyeon Seo, Dong-Hwa Choi, Yeon-Ju Nam, Daehee Hwang, Ayako Fukunaka, Yoshio Fujitani, Shigeyuki Yokoyama, Andrea Superti-Furga, Shiro Ikegawa, Tae Ryong Lee, Toshiyuki Fukada

**Affiliations:** 1 Bioscience Research Institute, Amorepacific Corporation R&D CenterYongin, Republic of Korea; 2 Division of Pathology, Department of Oral Diagnostic Sciences, School of Dentistry, Showa UniversityShinagawa, Japan; 3 Laboratory for Homeostatic Network, RIKEN Center for Integrative Medical SciencesYokohama, Japan; 4 Deutsches Rheuma-ForschungszentrumBerlin, Osteoimmunology, Berlin, Germany; 5 RIKEN Systems and Structural Biology CenterYokohama, Japan; 6 Division of Structural and Synthetic Biology, RIKEN Center for Life Science TechnologiesYokohama, Japan; 7 Department of Chemical Engineering, POSTECHPohang, Republic of Korea; 8 Laboratory for Bone and Joint Diseases, RIKEN Center for Integrative Medical SciencesTokyo, Japan; 9 Graduate School of Frontier Biosciences, Osaka UniversityOsaka, Japan; 10 Laboratory for Immune Regeneration, RIKEN Center for Integrative Medical SciencesYokohama, Japan; 11 Division of Integrated Life Science, Graduate School of Biostudies, Kyoto UniversityKyoto, Japan; 12 Gyeonggi Bio Center, Gyeonggi Institute of Science & Technology PromotionSuwon, Republic of Korea; 13 Center for Systems Biology of Plant Senescence and Life History, Institute for Basic ScienceDaegu, Republic of Korea; 14 Center for Beta-Cell Biology and Regeneration, Department of Metabolism and Endocrinology, Juntendo University Graduate School of MedicineTokyo, Japan; 15 RIKEN Structural Biology LaboratoryYokohama, Japan; 16 Department of Pediatrics, Centre Hospitalier Universitaire Vaudois, University of LausanneLausanne, Switzerland

**Keywords:** Proteasome, SCD-EDS, VCP, zinc transporter, ZIP13

## Abstract

The zinc transporter protein ZIP13 plays critical roles in bone, tooth, and connective tissue development, and its dysfunction is responsible for the spondylocheirodysplastic form of Ehlers-Danlos syndrome (SCD-EDS, OMIM 612350). Here, we report the molecular pathogenic mechanism of SCD-EDS caused by two different mutant ZIP13 proteins found in human patients: ZIP13^G64D^, in which Gly at amino acid position 64 is replaced by Asp, and ZIP13^ΔFLA^, which contains a deletion of Phe-Leu-Ala. We demonstrated that both the ZIP13^G64D^ and ZIP13^ΔFLA^ protein levels are decreased by degradation via the valosin-containing protein (VCP)-linked ubiquitin proteasome pathway. The inhibition of degradation pathways rescued the protein expression levels, resulting in improved intracellular Zn homeostasis. Our findings uncover the pathogenic mechanisms elicited by mutant ZIP13 proteins. Further elucidation of these degradation processes may lead to novel therapeutic targets for SCD-EDS.

## Introduction

Zinc (Zn) transporters are pivotal for Zn homeostasis, which is important for human health (Fukada & Kambe, 2011). Zn contributes to a variety of cellular functions and physiological events (Fukada *et al*, 2014), and impaired Zn regulation can cause a variety of diseases (Prasad, 1995; MacDonald, 2000; Lichten & Cousins, 2009; Fukada *et al*, 2011b; Ryu *et al*, 2011). One such disease is acrodermatitis enteropathica (AE), a pediatric disorder resulting from Zn deficiency. Patients with autosomal recessive AE have mutations in the *SLC39A4* gene (Wang *et al*, 2002; Dufner-Beattie *et al*, 2007), which encodes ZIP4, a membrane protein that mediates Zn influx across the cell membrane. A loss-of-function *SLC39A4* gene mutation in humans results in growth retardation, dermatitis, and hair loss (Wang *et al*, 2002; Dufner-Beattie *et al*, 2007). ZIP4 may also affect pancreatic cancer pathogenesis and progression (Li *et al*, 2007; Zhang *et al*, 2013), and intestinal integrity (Geiser *et al*, 2012). ZIP6 is reported to control metastasis (Yamashita *et al*, 2004; Hogstrand *et al*, 2013), ZIP7 is involved in the progression and proliferation of breast cancer cells (Taylor *et al*, 2007), and ZIP8 plays a key role in osteoarthritis-related cartilage destruction (Kim *et al*, 2014). Transient neonatal Zn deficiency is a disease related to the *SLC30A2* gene, which encodes the Zn efflux protein ZnT2. A heterozygous mutation in ZnT2 causes a low Zn concentration in mothers' milk, resulting in Zn deficiency in their breast-fed infants (Chowanadisai *et al*, 2006; Itsumura *et al*, 2013). ZnT8, which is expressed in pancreatic β cells, is essential for packaging insulin crystals (Bosco *et al*, 2010; Hardy *et al*, 2011), and variants in the *SLC30A8/ZnT8* gene are associated with an increased risk for type 2 diabetes (Xu *et al*, 2012; Tamaki *et al*, 2013).

The spondylocheirodysplastic form of Ehlers-Danlos syndrome (SCD-EDS, OMIM 612350), a genetic disorder of connective tissues, bones, and teeth, is also related to Zn imbalance (Fukada *et al*, 2008; Giunta *et al*, 2008; Warman *et al*, 2011; Byers & Murray, 2012). SCD-EDS patients show short stature, skeletal dysplasia of the spine, and clinical abnormalities of the hands and teeth, in addition to the common features of EDS such as skin and joint looseness. A mouse model of SCD-EDS, the *Slc39a13/Zip13-*deficient (*Zip13*-KO) mouse, has features similar to those of human patients, that is, abnormal development of the skin, bone, teeth, and craniofacial structures. (Fukada *et al*, 2008, 2011a; Munemasa *et al*, 2014). Molecular analyses revealed that the mesenchymal-originated cells from *Zip13*-KO mice have impaired BMP/TGF-β signaling, indicating that ZIP13 is critical for the development of hard and connective tissues (Fukada *et al*, 2008). By homozygosity mapping of Portuguese patients with SCD-EDS, we identified a pathogenic mutation (c.221G>A, G74D) in the *SLC39A13* gene (Fukada *et al*, 2008). The ectopic expression of the G74D ZIP13 mutant could not fully rescue *Zip13*-KO primary osteoblasts or dermal fibroblasts, indicating that G74D was a loss-of-function mutation (Fukada *et al*, 2008). This mutation was later renamed G64D, after identification of the *de facto* start codon 10 amino acids downstream from the conventional start codon, and its membrane topology was refined (Bin *et al*, 2011). Another mutant ZIP13 protein, in which phenylalanine–leucine–alanine (FLA) is deleted (ZIP13^ΔFLA^), was also reported in human SCD-EDS patients (Giunta *et al*, 2008). Characterization of the wild-type (WT) ZIP13 protein revealed that it is localized to the Golgi, possesses 8 putative transmembrane domains (TMs) with luminal N- and C-termini, and forms homo-dimers (Fukada *et al*, 2008; Bin *et al*, 2011), and its luminal loop was proposed to be responsible for Zn selection (Potocki *et al*, 2013). However, it remains unknown how the identified ZIP13 mutations lead to SCD-EDS.

Here, we demonstrate that both the ZIP13^G64D^ and ZIP13^ΔFLA^ proteins are rapidly degraded via the valosin-containing protein (VCP)-linked ubiquitin proteasome pathway, leading to an imbalance of intracellular Zn homeostasis. Furthermore, the protein expression levels and Zn homeostasis were recovered by inhibiting the proteasome machinery. This is the first demonstration of the mechanism by which these mutations cause the loss of ZIP13 function and SCD-EDS, and our findings may suggest potential therapies for treating this disease.

## Results

### The level of ZIP13^G64D^ protein is decreased in cultured cells

**Figure 1:**
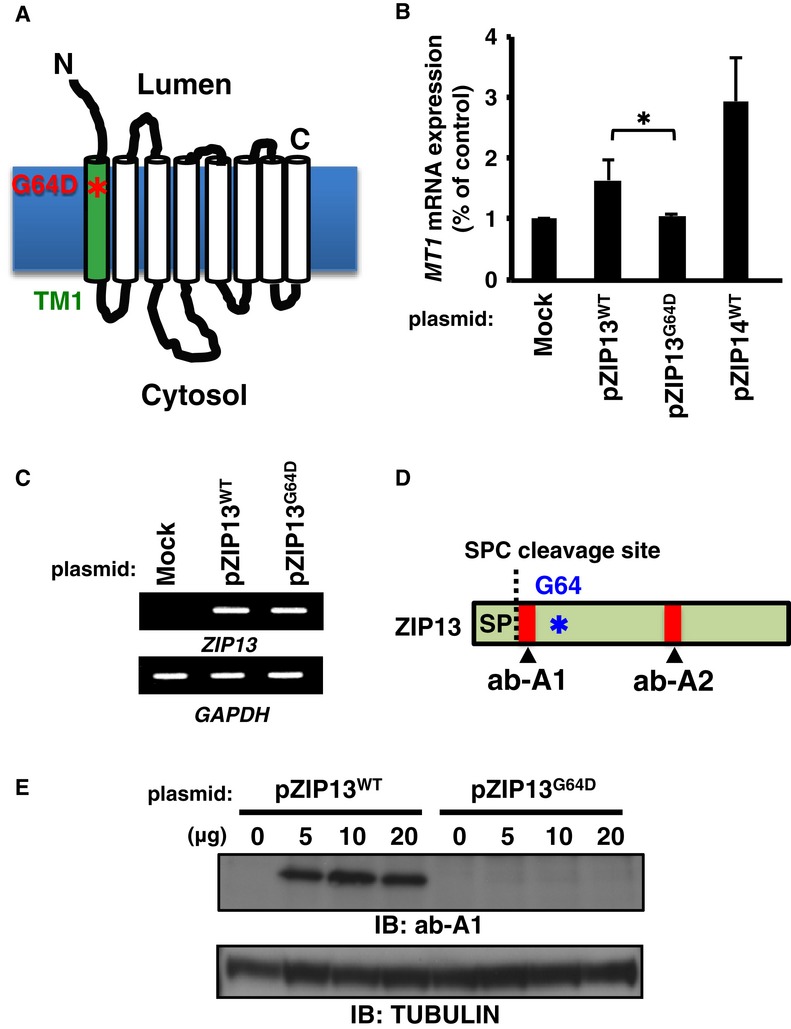
ZIP13 with the pathogenic G64D mutation shows a decreased protein expression level A  Location of the G64D mutation in ZIP13. Asterisk (*) indicates the G64D mutation. B   *Metallothionein 1* (*MT1*) expression. 293T cells transfected with the indicated DNA constructs were treated with 50 μM ZnSO_4_ for 6 h, and then, the *MT1* mRNA expression level was analyzed by RT-qPCR. Data are representative of three experiments and shown as mean ± s.e.m. (*P = 0.037). ZIP14^WT^ was included as a positive control. C   *ZIP13* transcript levels in 293T cells expressing wild-type or G64D mutant ZIP13. 293T cells were transfected with plasmids for ZIP13^WT^ or ZIP13^G64D^. Twenty-four hours later, RT–PCR was performed using primers for the indicated genes (Fukada *et al*, 2008). D   Schematic diagram showing the recognition sites of anti-ZIP13 antibodies. Asterisk (*) indicates the G64D mutation. SP, signal peptide; SPC, signal peptidase complex; ab-A1 and ab-A2 indicate anti-ZIP13 antibodies that recognize amino acids 23–35 of human ZIP13 and 184–201 of mouse ZIP13, respectively. E   ZIP13 protein levels in 293T cells expressing wild-type and G64D mutant ZIP13. Cell lysates were analyzed by Western blot (IB) using the ab-A1 antibody. Source data are available online for this figure.

To characterize the pathogenic ZIP13^G64D^ protein, in which a glycine at amino acid position 64 (G64), located within TM1, is replaced by aspartic acid (Fig 1A), we first introduced ZIP13^WT^- and ZIP13^G64D^-expressing plasmids into 293T cells. While ZIP13^WT^ increased the *Metallothionein 1* (*MT1*) gene expression (Fig 1B) reflecting an increased intracellular Zn level (Supplementary Fig S1), ZIP13^G64D^ did not, even though the *ZIP13*^*G64D*^ and *ZIP13*^*WT*^ transcript levels were equivalent (Fig 1C). In addition, the ZIP13 protein was barely detected by the anti-ZIP13 antibody ab-A1 (Fig 1D) in transiently ZIP13^G64D^-expressing 293T cells (Fig 1E). Similar results were obtained in HeLa cells stably expressing ZIP13^G64D^ (Supplementary Fig S2A). These findings suggested that the ZIP13^G64D^ protein was unstable, resulting in an imbalance of intracellular Zn homeostasis.

### The G64D mutation affects the stability of the ZIP13 protein

**Figure 2:**
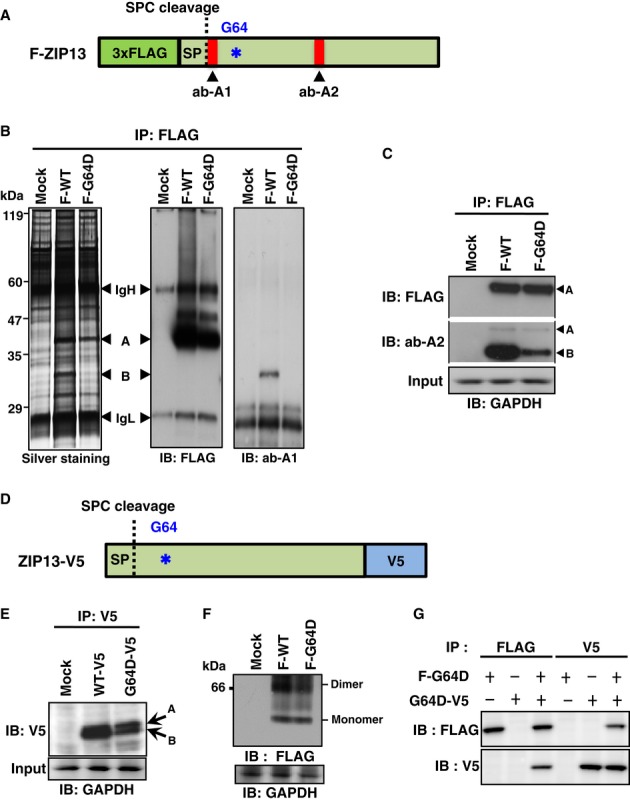
The pathogenic G64D mutation affects the stability of the SP-cleaved mature ZIP13 protein A   Schematic diagram of the N-terminally 3xFLAG-tagged ZIP13 protein (F-ZIP13). Asterisk (*) indicates the G64D mutation. SP, signal peptide; SPC, signal peptidase complex; ab-A1 and ab-A2: anti-ZIP13 antibodies. B   Protein expression of F-ZIP13 in 293T cells. N-terminally 3xFLAG-tagged wild-type (F-WT) and G64D mutant (F-G64D) ZIP13 proteins were immunoprecipitated (IP) with an anti-FLAG antibody, and then, the immunoprecipitates were analyzed by silver staining and Western blot using an anti-FLAG or anti-ZIP13 (ab-A1) antibody. IgH, heavy chain of IgG; IgL, light chain of IgG; A: SP-uncleaved immature ZIP13 protein; B: SP-cleaved mature ZIP13 protein. C   SP-cleaved mature ZIP13 protein was detected by ab-A2. A: SP-uncleaved immature ZIP13 protein; B: SP-cleaved mature ZIP13 protein. D   Schematic diagram of the C-terminally V5 epitope-tagged ZIP13 protein (ZIP13-V5). E   Protein expression of ZIP13-V5 in 293T cells. V5 epitope-tagged wild-type or G64D mutant ZIP13 protein (WT-V5 or G64D-V5) was immunoprecipitated using an anti-V5 antibody, and then, the immunoprecipitate was analyzed by Western blot using an anti-V5 antibody. A: SP-uncleaved immature ZIP13 protein; B: SP-cleaved mature ZIP13 protein. F   Dimer formation assay. The dimer formation of ZIP13 was analyzed by blue native-PAGE using the lysates of 293T cells expressing F-WT or F-G64D. F   Monomer–monomer interaction assay. 293T cells were co-transfected with expression plasmids for F-G64D and G64D-V5 ZIP13, followed by immunoprecipitation with the indicated antibodies. Western blotting analysis was performed with either an anti-V5 or anti-FLAG antibody. Source data are available online for this figure.

We previously identified the signal peptide (SP) of the ZIP13 protein (Fig 1D) (Bin *et al*, 2011). SP is cleaved to yield the “mature” protein, that is, the functional protein with the correct intracellular distribution. To determine whether the G64D mutation affects the level of the mature ZIP13 or the SP-uncleaved “immature” protein, we generated two anti-ZIP13 antibodies: one against a synthetic peptide corresponding to an internal sequence (amino acids 23–35) in human ZIP13, proximal to the signal peptidase complex (SPC) cleavage site (ab-A1) and another against amino acids 184–201 of mouse ZIP13 (ab-A2) (Figs 1D and 2A). When the lysates of 293T cells expressing N-terminally 3xFLAG-tagged wild-type ZIP13 (Fig 2A) were immunoprecipitated using anti-FLAG antibody, separated by SDS–PAGE, and subjected to silver staining, two unique bands were observed with molecular weights between 29 and 47 kDa (band-A and band-B) (Fig 2B, left). In contrast, when cells expressing mutant ZIP13 (F-G64D) were treated similarly, band-B was severely decreased while band-A remained (Fig 2B, left). Western blot using an anti-FLAG antibody revealed that band-A contained FLAG and was therefore the SP-uncleaved, immature ZIP13 protein (Fig 2B, middle). Band-B was recognized in the F-WT sample by ab-A1 (Fig 2B, right), but not by the anti-FLAG antibody (Fig 2B, middle), indicating that it was the SP-cleaved, mature ZIP13^WT^ protein. No bands were detected by the ab-A1 antibody in the F-G64D sample (Fig 2B, right), indicating that the SP-cleaved ZIP13^G64D^ mature protein was specifically decreased in the cells. Western blot with the ab-A2 antibody revealed band-B at a lower position, most likely corresponding to the SP-cleaved, mature ZIP13 protein (Fig 2C, middle), and the amount of band-B yielded by the expression plasmid for F-G64D was markedly decreased (Fig 2C, middle). Furthermore, when the lysates from cells expressing a C-terminally V5 epitope-tagged ZIP13 (ZIP13-V5) (Fig 2D) were subjected to Western blot with an anti-V5 antibody, the V5-tagged mutant (G64D-V5) levels were lower (Fig 2E and Supplementary Fig S2A), similar to the results with F-G64D (Fig 2B). While immunoprecipitation analysis showed the same two bands in both the wild-type (WT-V5) and G64D-V5 samples (Fig 2E, band-A and band-B), the G64D-V5-expressing cells contained a reduced amount of band-B, indicating that the expression of SP-cleaved G64D mature protein was greatly reduced in these cells.

Since ZIP13 protein forms a homo-dimer (Bin *et al*, 2011) and the G87R mutation in the zinc transporter ZnT2 is reported to cause neonatal zinc deficiency due to a dominant-negative effect on its homo-dimerization (Lasry *et al*, 2012), we next examined whether the G64D mutation affects the oligomeric state of the ZIP13 protein. Blue native-PAGE analysis of lysates from F-ZIP13-expressing 293T cells showed a lower expression of F-G64D than F-WT, but the F-G64D apparently still formed dimers similar to F-WT (Fig 2F). We further evaluated the monomer–monomer interaction between ZIP13^G64D^ proteins in 293T cells that were co-transfected with plasmids encoding F-G64D and G64D-V5, followed by immunoprecipitation with anti-FLAG or anti-V5 antibodies. Western blotting analysis clearly showed that F-G64D and G64D-V5 formed a complex (Fig 2G). Taken together, these results indicated that the loss of function of the G64D mutation was mainly attributable to a large reduction in the quantity of the mature ZIP13 protein, rather than to a disruption in ZIP13's ability to form a complex due to a change in its biochemical characteristics.

### Proteasome-dependent pathways are involved in the degradation of ZIP13^G64D^ protein

**Figure 3:**
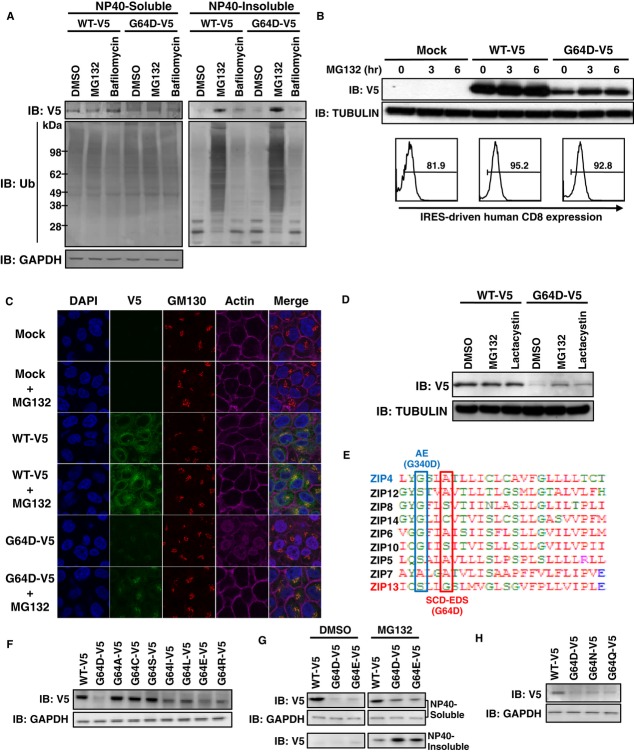
ZIP13^G64D^ protein is readily degraded by a proteasome-dependent mechanism A   Proteasome inhibitor treatments. 293T cells were transfected with WT-V5 or G64D-V5 ZIP13 and treated with 10 μM MG132 or 1 μM bafilomycin for 6 h. Cells were lysed in 1% NP-40 and then separated into soluble and insoluble fractions. Western blotting analysis was performed with an anti-V5 or anti-ubiquitin antibody. B   HeLa cells expressing WT-V5 or G64D-V5 (Supplementary Fig S2A) were treated with 10 μM MG132 for the indicated periods. (Upper) Total cell lysates were analyzed by Western blot using an anti-V5 antibody. (Lower) The hCD8 levels indicate the amount of transfected plasmid DNA (pMX-WT-IRES-hCD8 or pMX-G64D-IRES-hCD8). Cells were analyzed by flow cytometry using APC-conjugated anti-hCD8 antibody. Histograms were gated on hCD8-positive cells. C   Confocal images of ZIP13. HeLa cells stably expressing the indicated proteins were treated with or without MG132. Nuclei (blue), ZIP13 (green), Golgi (red), and actin (magenta) were stained with DAPI, anti-V5 antibody, anti-GM130 antibody, and Phalloidin, respectively. D   HeLa cells stably expressing the indicated proteins were treated with proteasome inhibitors 10 μM MG132 or 1 μM lactacystin for 6 h, followed by Western blot of whole-cell lysates using an anti-V5 antibody. E   Location of pathogenic mutations in TM1. Amino acid alignment of the TM1 of human ZIP family members. Red: hydrophobic amino acids; blue: acidic amino acids; magenta: basic amino acids; green: hydrophilic amino acids. AE (G340D): amino acid substitution in ZIP4 of AE patients; SCD-EDS (G64D): amino acid substitution in ZIP13 of SCD-EDS patients. F   The 64th amino acid influences ZIP13 protein stability. C-terminally V5-tagged ZIP13 expression plasmids with a mutation at position 64 were transfected into 293T cells and analyzed by Western blot using an anti-V5 antibody. G   Mutant ZIP13 constructs with an acidic amino acid at position 64. 293T cells were transfected with C-terminally V5-tagged ZIP13 expression plasmids, treated with MG132, lysed in NP-40, separated into soluble and insoluble fractions, and analyzed using an anti-V5 antibody. H   Mutant ZIP13 constructs in which glycine 64 was replaced with asparagine (G64N) or glutamine (G64Q). Total cell lysates were analyzed by Western blot using an anti-V5 antibody. Source data are available online for this figure.

Given that the expression level of ZIP13^G64D^ protein but not its mRNA was reduced, it was likely that a protein degradation pathway was involved. To address this possibility, we expressed ZIP13-V5 (Fig 2D) in 293T cells, followed by treatment with MG132, an inhibitor of proteasome-dependent degradation pathways, or bafilomycin, an inhibitor of lysosome-dependent degradation pathways (Lee & Goldberg, 1998; Ishidoh & Kominami, 2002). The cells were then lysed by a detergent-containing buffer, and the lysates were separated into soluble and insoluble fractions by brief centrifugation and subjected to Western blotting analysis. The protein level of G64D-V5 in the NP40-detergent-soluble fraction was lower than that of WT-V5 (Fig 3A, left), similar to the result using non-tagged ZIP13s (Fig 1E). While bafilomycin had no apparent effect on the protein expression patterns, MG132 preferentially increased the amount of WT-V5 and G64D-V5 protein in the NP40-detergent-insoluble fraction, which contained numerous ubiquitinated proteins, and in which the level of G64D-V5 was greater than that of WT-V5 (Fig 3A, right). These findings indicated that ZIP13 is normally degraded by a proteasome-dependent pathway and that the G64D mutation alters the protein's properties so that more of it accumulates in the detergent-insoluble fraction.

To confirm that the ZIP13^G64D^ protein was degraded via a proteasome-dependent pathway, we repeated the experiment using a different cell line. HeLa cell lines stably expressing WT-V5 or G64D-V5 were established by blasticidin selection. Clones containing similar amounts of transfected cDNA were selected by monitoring their internal ribosome entry site (IRES)-driven human CD8 expression (Fig 3B, lower, and Supplementary Fig S2C), then treated with the proteasome inhibitor MG132. Western blotting analysis showed that MG132 treatment led to an increase in the G64D-V5 protein over time (Fig 3B, upper), accumulating it most likely in the Golgi (Fig 3C), where ZIP13 is normally localized (Fukada *et al*, 2008). Furthermore, treatment with lactacystin, another proteasome inhibitor, upregulated the G64D-V5 protein expression (Fig 3D). The ZIP13 homodimers were also increased when MG132 was applied (Supplementary Fig S3). These findings suggested that the G64D protein enters a proteasome-dependent degradation pathway.

Amino acid alignment showed that ZIP family members share a small and neutral amino acid at the site corresponding to the 64th position of ZIP13 (Fig 3E). To determine how the amino acid composition at this position affects protein stability, we next substituted different amino acids at the 64th position, using a variety of approaches. Replacement of G64 with an amino acid containing a small side chain, such as alanine (G64A), cysteine (G64C), or serine (G64S), caused little change in the protein expression level from that of wild-type ZIP13 (Fig 3F). However, the replacement with an amino acid containing a large side chain, isoleucine (G64I) or leucine (G64L), or with the basic amino acid arginine (G64R) significantly reduced the protein level, although not to the same extent as with aspartic acid, an acidic amino acid (G64D) (Fig 3F). We thus hypothesized that the acidic side chain in G64D interferes with the stability of the ZIP13 protein. To address this possibility, we replaced G64 with another acidic amino acid, glutamic acid (G64E), and observed a severe decrease in the ZIP13^G64E^ protein level, comparable to ZIP13^G64D^ (Fig 3F and G). Notably, the transcript levels of these mutants were all comparable to that of wild type (Supplementary Fig S4A), and MG132 treatment caused ZIP13^G64E^ protein to be recovered in the insoluble fraction, similar to ZIP13^G64D^ protein (Fig 3G). The replacement of G64 with asparagine (G64N) or glutamine (G64Q) also reduced the protein level, but to a lesser extent than G64D (Fig 3H), while the transcription level was similar to wild-type cells (Supplementary Fig S4B). Based on these findings, we concluded that a small and neutral amino acid at the 64th position is critical for the stability of the ZIP13 protein. The replacement of G64 with an amino acid having a large or basic side chain caused its protein level to decrease, and acidity at the 64th position was fatal to the ZIP13 protein, leading to its clearance by the proteasome-dependent (20S proteasome-independent: Supplementary Fig S5) degradation pathway.

### Pathogenic ZIP13 proteins are degraded by the ubiquitination-dependent pathway

**Figure 4:**
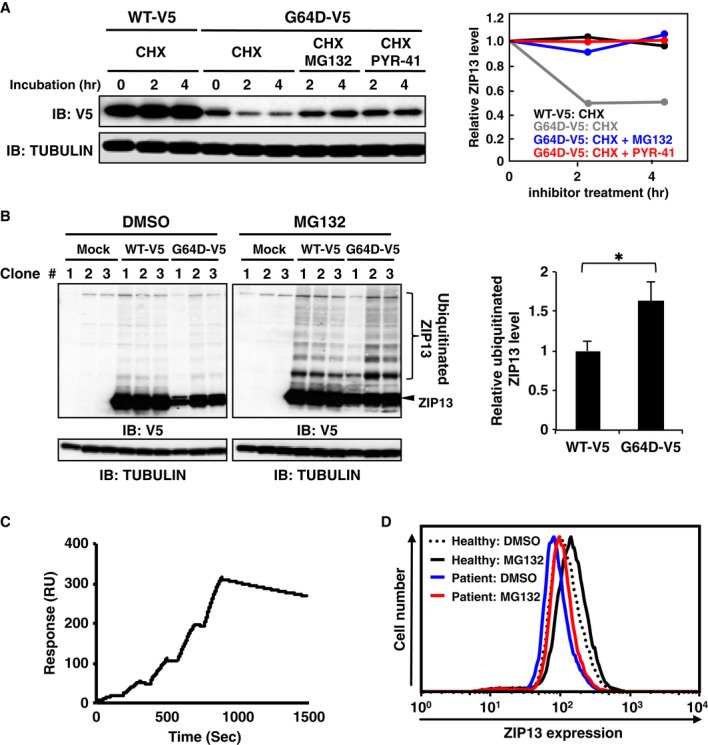
ZIP13^G64D^ protein is degraded by a ubiquitination-dependent pathway A   Treatment with PYR-41, a ubiquitin E1 inhibitor, suppressed the downregulation of ZIP13^G64D^ protein in the presence of cycloheximide (CHX). HeLa cells stably expressing WT-V5 or G64D-V5 were treated with 10 μM MG132 or 10 μM PYR-41 together with CHX for the indicated times. Total cell lysates were subjected to Western blotting analysis with an anti-V5 antibody. Right panel shows the relative expression levels of ZIP13 proteins. Data are representative of two independent experiments. B   HeLa cells stably expressing WT-V5 or G64D-V5 (Supplementary Fig S2A) were treated with 10 μM MG132 for 6 h. The cell lysates were analyzed by Western blot using an anti-V5 antibody. The ubiquitinated/non-ubiquitinated G64D protein ratio was upregulated compared to that of wild type (right panel). Data are shown as mean ± s.e.m. (*P = 0.036). C   Single cycle kinetic analysis of ZIP13 protein binding to the amine-coupled antibody 35B11 on a Biacore sensor tip. Solution-phase ZIP13-35B11 binding was measured by surface plasmon resonance (BIAcore). A representative BIAcore sensorgram shows the response over time (resonance units [RU]) during the binding of purified recombinant human ZIP13 protein to immobilized 35B11 antibodies. Purified human ZIP13 protein at concentrations of 25, 50, 100, 200, and 400 nM was added at 0, 190, 380, 570, and 760 s, respectively. The graph is representative of four independent experiments. D   Intracellular flow cytometric analysis of the endogenous ZIP13 expression in a healthy female donor or female SCD-EDS patient. Cultured primary human fibroblasts were treated with DMSO or 10 μM MG132 for 6 h. After fixation and permeabilization, the cells were stained with the monoclonal antibody 35B11, followed by goat anti-mouse Alexa 488. Data are representative of two independent experiments. Similar results were obtained in a healthy male donor and male SCD-EDS patient. Source data are available online for this figure.

To determine whether the ZIP13^G64D^ protein was ubiquitinated, 6 × histidine-tagged mono-ubiquitin was co-expressed with ZIP13^WT^-V5 or ZIP13^G64D^-V5 in 293T cells; then, the ubiquitin-containing proteins were purified using Ni-NTA agarose under denaturing conditions. Ubiquitinated ZIP13^WT^ or ZIP13^G64D^ protein was elevated in the MG132-treated samples (Supplementary Fig S6). Consistent with this finding, cotreatment with PYR-41 (a ubiquitin-activating enzyme E1 inhibitor) and the protein synthesis inhibitor cyclohexamide (CHX) suppressed the decrease in mutant ZIP13 protein expression in HeLa cells (Fig 4A). In addition, we noted an increase in the slowly migrating ubiquitinated wild-type ZIP13 protein after MG132 treatment (Fig 4B, left) and that the ubiquitinated/non-ubiquitinated G64D protein ratio was significantly higher than that of wild type (Fig 4B, right). These findings suggested that the wild-type ZIP13 protein is turned over by the ubiquitin proteasome pathway, but the G64D mutant is more extensively degraded by this pathway.

Next, we investigated whether these results were applicable to cells from SCD-EDS patients. We first generated the monoclonal anti-human ZIP13 antibody 35B11 clone using the “liposome immunization” system and the three-step screening method (Hino *et al*, 2013). This method is useful for producing antibodies that recognize the tertiary structure of a membrane protein with high affinity (Hino *et al*, 2013). The 35B11 clone was confirmed to bind the purified ZIP13 protein, assessed by surface plasmon resonance (SPR) experiments (Fig 4C). Sensorgrams fitted to a 1:1 binding model using the Biacore T200 Evaluation Software yielded the following average kinetic constants: *k*_a_, 1.34 ± 0.04 × 10^4^ M^−1^ s^−1^; *k*_d_, 2.59 ± 0.3 × 10^−4^ s^−1^; *K*_*D*_, 19.3 ± 2.7 nM. Flow cytometric analyses using 35B11 demonstrated that the level of ZIP13^G64D^ protein was significantly reduced compared to ZIP13^WT^ protein in HeLa stable lines (Supplementary Fig S7), confirming that this antibody was also useful for detecting the cellular ZIP13 proteins. We next prepared primary cultured fibroblasts from the biopsies of healthy donors and SCD-EDS patients who expressed the ZIP13^G64D^ protein and compared the ZIP13 protein levels. Consistent with the results in cell lines, the expression level of ZIP13 protein was decreased in the cells from patients compared to those from healthy donors (Fig 4D, blue line versus dotted line). Importantly, MG132 treatment of the SCD-EDS patient cells increased the total ZIP13^G64D^ protein expression to the level of healthy donors (Fig 4D, red line versus dotted line), indicating that the pathogenic G64D mutation of ZIP13 in SCD-EDS patients causes degradation of the functional protein by the proteasome-dependent pathway.

**Figure 5:**
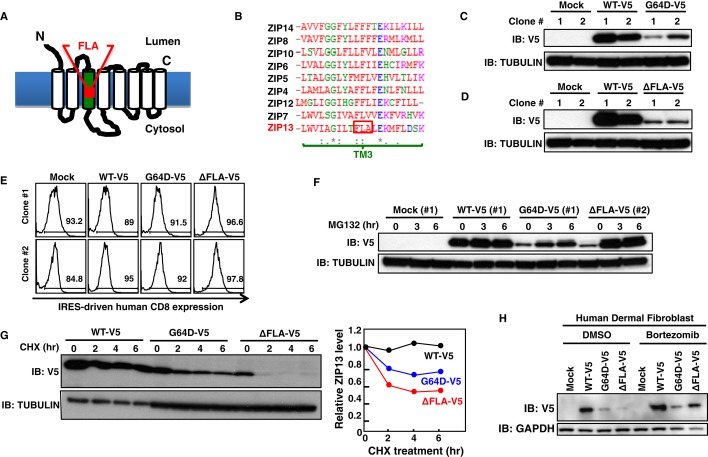
ZIP13^ΔFLA^ protein is degraded by a proteasome-dependent pathway A   Location of the ΔFLA mutation (deletion of phenylalanine–leucine–alanine in TM3) in ZIP13. B   Amino acid alignment of the TM3 of human ZIP family members. Amino acids conserved in all of the indicated zinc transporters (*), conserved substitutions (:), semi-conserved substitutions (.). Red: hydrophobic amino acids; blue: acidic amino acids; magenta: basic amino acids; green: hydrophilic amino acids. C   Protein expression level of G64D-V5 in 293T stable lines. The cell lysates of two representative clones stably expressing WT-V5 or the G64D-V5 mutant were analyzed by Western blot using an anti-V5 antibody. D   Protein expression level of ΔFLA-V5 in 293T stable lines. The cell lysates of two representative clones stably expressing WT-V5 or the ΔFLA-V5 mutant were analyzed by Western blot using an anti-V5 antibody. E   The hCD8 expression levels in 293T stable lines, as an indicator of the amount of transfected plasmid DNA (pMX-WT-IRES-hCD8, pMX-G64D-IRES-hCD8, or pMX-ΔFLA-IRES-hCD8). Two representative clones stably expressing WT-V5 or the G64D-V5 or ΔFLA-V5 mutant were analyzed by flow cytometry using an APC-conjugated anti-hCD8 antibody. Histograms were gated on hCD8-positive cells. F   Recovery of mutant ZIP13 protein expression by MG132 treatment. Representative 293T clones stably expressing WT-V5 (#1), G64D-V5 (#1), or ΔFLA-V5 (#2), were treated with 10 μM MG132 for the indicated times, followed by Western blotting analysis with an anti-V5 antibody. G   Posttranslational degradation of mutant ZIP13 proteins. HeLa clones stably expressing WT-V5, G64D-V5, or ΔFLA-V5 were treated with 10 μM CHX for the indicated times. Total cell lysates were analyzed by Western blot using an anti-V5 antibody (upper). Right graph shows the relative expression level of ZIP13 proteins over time. Data are representative of three independent experiments. H   Protein expression level of the SCD-EDS pathogenic mutants in human fibroblasts in the presence of bortezomib. Human dermal fibroblasts transiently expressing ZIP13 mutants were treated with 10 nM bortezomib for 6 h, followed by Western blotting analysis using an anti-V5 antibody. Source data are available online for this figure.

We also studied the effect on protein levels of another ZIP13 mutation (Giunta *et al*, 2008), in which three amino acids (phenylalanine–leucine–alanine: FLA) in TM3 are deleted as the result of a frame shift (ZIP13^ΔFLA^, Fig 5A and B). The ZIP13^ΔFLA^ protein expression was also reduced although it was more unstable than the ZIP13^ΔG64D^ protein, and failed to increase the intracellular Zn level in 293T cells and in HeLa cells stably introduced with its expression plasmid (Fig 5C–G, Supplementary Figs S1 and S2). Moreover, ZIP13^ΔFLA^ protein was readily restored after MG132 treatment (Fig 5F), indicating that it was degraded by the proteasome-dependent pathway as well as the ZIP13^G64D^ protein.

Bortezomib is a therapeutic proteasome inhibitor that acts by reversibly binding to the catalytic site of the 26S proteasome (Teicher *et al*, 1999; Lightcap *et al*, 2000). Using the human dermal fibroblast and 293T cells, we found that bortezomib restored the ZIP13^G64D^ and ZIP13^ΔFLA^ mutant protein levels (Fig 5H and Supplementary Fig S8A and B), accompanied by normalization of the intracellular Zn level (Supplementary Fig S8C) as the MG132 treatment does (Supplementary Fig S9). These observations suggested that 26S proteasome inhibitors could restore the impaired intracellular Zn homeostasis by the ZIP13 mutants; thus, the manipulation of 26S proteasome activity by inhibitory compounds might be a therapeutic approach for SCD-EDS caused by pathogenic mutant ZIP13 proteins.

### VCP is involved in the degradation of the mutant ZIP13 proteins

**Figure 6:**
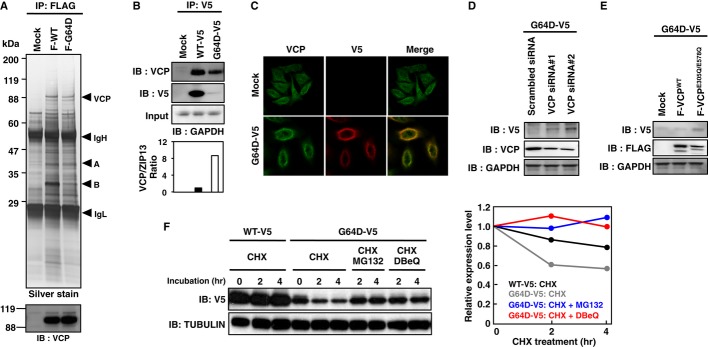
The mutant ZIP13 protein is degraded through a VCP-dependent mechanism A   Identification of VCP/Cdc48/p97 as a ZIP13-associating protein. Whole-cell lysates from 293T cells transfected with FLAG-tagged ZIP13 were immunoprecipitated with an anti-FLAG antibody, followed by SDS–PAGE and silver staining. Unique bands were cut out and analyzed by TOF/MASS to identify the proteins. A protein band near 88 kDa was determined to be VCP/Cdc48/p97. VCP was also detected by Western blot using an anti-VCP antibody (lower). IgH: heavy chain of IgG; IgL: light chain of IgG; A: SP-uncleaved immature ZIP13 protein; B: SP-cleaved mature ZIP13 protein. B   VCP binds to ZIP13. Whole-cell lysates from 293T cells transfected with expression plasmids for V5-tagged ZIP13 proteins were immunoprecipitated with an anti-V5 antibody, followed by SDS–PAGE. VCP and ZIP13 proteins were detected by Western blot using anti-VCP and anti-V5 antibodies, respectively. The VCP/ZIP13 ratio was analyzed using ImageJ software (http://rsbweb.nih.gov/ij/download.html) (bottom). C   Confocal images of VCP in HeLa cells stably expressing G64D-V5. VCP (green) and G64D-V5 (red) were stained with anti-V5 and anti-VCP antibodies, respectively. D   Effect of VCP siRNA on the protein expression of G64D-V5 in HeLa cells. VCP siRNA was transfected into HeLa cells stably expressing G64D-V5. Seventy-two hours posttransfection, the cells were harvested and subjected to Western blotting analysis using anti-V5 or anti-VCP antibodies. E   Effect of a dominant-negative form of VCP on the protein expression of G64D-V5 in HeLa cells. 3xFLAG-tagged wild-type VCP^WT^ and dominant-negative VCP^E305Q/E578Q^ were transfected into HeLa cells stably expressing G64D-V5. Twenty-four hours later, the cells were lysed and then subjected to Western blotting analysis with anti-V5 or anti-FLAG antibodies. F   Effect of a VCP inhibitor, DBeQ on the protein expression of G64D-V5 in HeLa cells. HeLa cells stably expressing WT-V5 or G64D-V5 were treated with 10 μM MG132 or 10 μM DBeQ together with CHX for the indicated times. The cell lysates were subjected to Western blotting analysis with an anti-V5 antibody. Right graph shows the relative expression level of ZIP13 proteins. Data are representative of two independent experiments. Source data are available online for this figure.

To further elucidate the molecular mechanisms involved in normal and pathogenic ZIP13 homeostasis, we isolated ZIP13-associated molecules by immunoprecipitation. Of these, we identified VCP/Cdc48/p97 by mass spectrometric analysis (Fig 6A). VCP belongs to the AAA superfamily, which mediates multiple functions, including the ubiquitination-dependent proteasome system (Ye *et al*, 2001, 2004; Richly *et al*, 2005). In addition to ZIP13^WT^, VCP bound to and co-localized with the mutant ZIP13^G64D^ protein (Fig 6A–C). Intriguingly, more VCP was associated with ZIP13^G64D^ than with ZIP13^WT^ (Fig 6B, lower), indicating that the VCP protein might preferentially interact with the pathogenic ZIP13^G64D^ protein. To understand VCP's role in the degradation of the mutant ZIP13 protein, we knocked down VCP by siRNAs or suppressed its function by expressing a dominant-negative form of VCP. VCP siRNAs reduced the protein level of the endogenous VCP (Fig 6D, middle) and restored the protein level of ZIP13^G64D^ (Fig 6D, upper). Furthermore, the ectopic expression of dominant-negative VCP, F-VCP^E305Q/E578Q^, restored the protein level of ZIP13^G64D^ (Fig 6E). In addition, a VCP inhibitor DBeQ (Chou *et al*, 2011) could suppress the decay of the ZIP13^G64D^ protein (Fig 6F). These findings suggested that the VCP-linked proteasome-dependent pathway is involved in the normal steady-state turnover of wild-type ZIP13 and is critical for the clearance of the pathogenic mutant ZIP13 protein.

## Discussion

**Figure 7:**
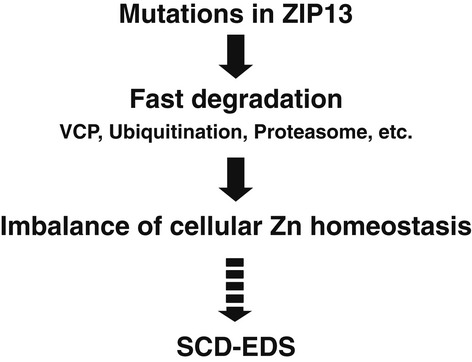
Pathogenic mutations in ZIP13 result in its rapid reduction and zinc imbalance, leading to SCD-EDS Pathogenic mutations cause the mutant ZIP13 proteins to enter the VCP-linked ubiquitin proteasome degradation pathway, resulting in reduced protein expression levels and imbalance of cellular Zn homeostasis.

In the present study, we investigated the molecular pathogenic basis of the mutant ZIP13 proteins ZIP13^G64D^ and ZIP13^ΔFLA^, which are responsible for SCD-EDS, to determine how these mutations lead to the loss of ZIP13 function. We demonstrated that the degradation of functional ZIP13 proteins by the VCP-linked ubiquitin proteasome pathway is the major pathogenic consequence of these mutations and that the resultant disturbance of intracellular Zn homeostasis can cause SCD-EDS (Fig 7).

In both the ZIP13^G64D^ and ZIP13^ΔFLA^ proteins, the pathogenic mutation occurs in a TM domain (Fukada *et al*, 2008; Giunta *et al*, 2008). TM domains are generally composed of hydrophobic amino acids, which interact with lipids and often form a helix (Singer & Nicolson, 1972). The Gly-X-X-Gly motif, a well-known motif found in helices, plays a critical role in helix-helix packing (Dohan & Carrasco, 2003; Kim *et al*, 2004; Munter *et al*, 2010). In this motif, the first and last glycine can be replaced by another amino acid with a small side chain (alanine, serine, or cysteine) (Dohan & Carrasco, 2003; Kim *et al*, 2004; Munter *et al*, 2010). In the case of ZIP13^G64D^, we demonstrated that replacing glycine 64, which is within a Ser-X-X-Gly motif, with a bulky amino acid with a large side chain (leucine, isoleucine, glutamic acid, or arginine) reduced the protein expression level, but replacement with alanine, serine, or cysteine did not (Fig 3F), revealing that an amino acid with a small side chain at position 64 is important for ZIP13's protein stability. In the proton-coupled folate transporter (PCFT), a Gly-X-X-Gly motif is proposed to provide conformational flexibility due to the lack of a side chain and was shown to be involved in PCFT's stability (Zhao *et al*, 2012). In our study, only the substitution of glycine 64 with an acidic amino acid, glutamic acid (G64E mutation), reduced the mutant ZIP13 protein level as severely as the G64D mutation, indicating that not only the size of the side chain, but also its negative charge may be important for the loss of G64D function. Reports on another Zn-imbalance disorder, AE, reveal a variety of mutations in the human *ZIP4* gene from these patients (Andrews, 2008). These mutations include G340D, G384R, G643R, and L382P in Gly-X-X-Gly motif-like and leucine zipper-like regions; of these, G384R, G643R, and L382P reduce the protein level, although the mechanism underlying this decrease is not fully known (Wang *et al*, 2002). Intriguingly, the improper posttranslational modification of ZIP4's N-terminal ectodomain is observed in some cases (Kambe & Andrews, 2009). When Zn is deficient, the N-terminal ectodomain of the mouse ZIP4 protein is cleaved, and the resulting protein accumulates on the plasma membrane to up-regulate Zn import. The G340D, G384R, and G643R mutants of ZIP4 show decreased ectodomain cleavage in response to Zn deficiency. In contrast to ZIP4, ZIP13 does not possess an ectodomain cleavage site at its N-terminus (Kambe & Andrews, 2009; Bin *et al*, 2011), implying that a mutation in ZIP13's Gly-X-X-Gly motif induces loss of function by a mechanism distinct from that elicited by ZIP4 mutations. The G340D ZIP4 mutation in AE patients occurs in a Gly-X-X-Gly motif in TM1, comparable to the G64 position in ZIP13 (Fig 3E), consistent with the importance of this motif in ZIP family members. Our finding that the FLA deletion in TM3 caused the rapid proteasome-dependent degradation of ZIP13 (Fig 5 and Supplementary Fig S2) suggests that SCD-EDS by the FLA deletion is also initially caused by a reduction in functional ZIP13 protein (Jeong *et al*, 2012).

Our biochemical analyses demonstrated that the pathogenic mutations caused the ZIP13 protein to be unstable and enter a proteasome-dependent degradation pathway (Figs 3, 4, 5, 6 and 7). In the case of ZIP4, elevated Zn promotes the endocytosis and degradation of the ZIP4 protein. In this process, lysines near the histidine-rich cluster between TM3 and TM4 of ZIP4 are modified by ubiquitination (Mao *et al*, 2007). We detected ubiquitinated ZIP13 protein (Fig 4B), although ZIP13 does not contain a typical histidine-rich cluster between TM3 and TM4, nor any other histidine clusters (Bin *et al*, 2011). We also found that VCP associates with either wild-type or mutant ZIP13 proteins, even though it preferentially interacts with the mutant ZIP13, suggesting that the VCP–ZIP13 interaction is important for both the normal steady-state turnover of wild-type ZIP13 and the clearance of ZIP13 proteins containing critical mutations (Fig 6). VCP was originally identified as a valosin-containing protein in pigs (Koller & Brownstein, 1987) and has roles in nucleus reformation, membrane fusion, protein quality control, autophagy, and other cellular processes (Latterich *et al*, 1995; Bukau *et al*, 2006; Ramadan *et al*, 2007; Buchan *et al*, 2013). VCP may mediate the retro-translocation of ZIP13 from the membrane into the cytosol before or after ZIP13's ubiquitination, along with various chaperones and ubiquitin-binding proteins that help deliver it to the proteasome for degradation (Ye *et al*, 2001, 2004; Richly *et al*, 2005). In addition to VCP, heat-shock proteins may be involved, because we found that the treatment of 17AAG, an HSP90 inhibitor, also restored the expression level of ZIP13^G64D^ protein (Supplementary Fig S10), supporting the idea that various molecules take part in ZIP13's degradation. The precise mechanism for ZIP13's degradation awaits future studies, but clues may lie in the identification of proteins that bind the extra/intracellular loops of ZIP13. Although mutated proteins sometimes induce ER stress before being degraded (Vidal *et al*, 2011), the expression level of ER-stress-responsive molecules was comparable between the cells expressing ZIP13^WT^ and the pathogenic mutants (Supplementary Fig S11), indicating that ER stress might not significantly participate in the pathogenic process of mutant ZIP13 proteins.

Importantly, our results lend credence to the potential use of proteasome inhibitors in clinical investigations of SCD-EDS and its therapeutics (Figs 3, 4, 5, and Supplementary Figs S8 and S9). We also found that VCP inhibitor improved the protein level of the pathogenic ZIP13 mutants (Fig 6F), further supporting the therapeutic potential of compounds targeted to proteasome pathways. Cystic fibrosis is a genetic disease caused by mutations in the cystic fibrosis transmembrane conductance regulator (CFTR). Ninety percent of the patients have a ΔF508 mutation, which prevents proper folding and processing of the CFTR protein; as a result, little of the mutant protein reaches the cell surface (Rommens *et al*, 1988; Riordan *et al*, 1989; Ward *et al*, 1995). Much research has focused on elucidating the folding, trafficking, and degradation properties of CFTR pathogenic mutants, and on developing drugs that are either “potentiators” of CFTR itself or “correctors” of its degradation pathway (Wang *et al*, 2008; Becq, 2010; Gee *et al*, 2011). VX-809 is the latest CFTR drug. It was obtained from a screen as a compound that reduces degradation of the ΔF508 mutant protein and increases CFTR accumulation on the cell surface and is currently in clinical trials (Van Goor *et al*, 2011). Another mutation, G551D, which accounts for about 5% of the cystic fibrosis patients, does not affect the protein's trafficking, but prohibits proper channel gating. Kalydeco (VX-770) was developed to treat cystic fibrosis patients carrying the G551D mutation (Van Goor *et al*, 2009; Accurso *et al*, 2010). It acts as a “potentiator” to open the gate of CFTR for proper chloride transport (Rowe & Verkman, 2013). In the case of SCD-EDS patients, therapeutic strategies analogous to those used to treat cystic fibrosis, as either molecular “potentiators” or “correctors”, may be effective depending on the functional consequences of the mutation. Moreover, we cannot exclude the possible involvement of another degradation pathway or translational defects of the ZIP13 mutants as a consequence of the mutation, given that the ZIP13^ΔFLA^ protein level recovered much more than the ZIP13^G64D^ protein level after MG132 treatment (Fig 5F and H) although the ZIP13^ΔFLA^ protein was more unstable than the ZIP13^G64D^ protein (Fig 5G). Future investigations of the molecular details underlying the degradation of G64D and ΔFLA mutants, and of the protein structure and homeostasis of ZIP13, will provide a framework to develop potential treatments for SCD-EDS and for the related metabolic diseases since ZIP13 is also implicated in adipose and muscle tissues homeostasis (Fukada *et al*, 2008). In this regard, mutant *ZIP13* gene knock-in mice could be useful animal models to develop therapeutics for SCD-EDS, and the development of Zn transport assay system using proteoliposomes with purified ZIP13 proteins may also facilitate further understandings of the physio-pathogenesis of ZIP13.

Taken together, we have gained insight into the mechanism underlying the loss of function of ZIP13 mutants in SCD-EDS patients (Fig 7). This mechanism involves the disruption of Zn regulation through a reduction of the ZIP13 protein level via the VCP-linked ubiquitin and proteasome-dependent degradation pathway. We found that conserved amino acid(s) in TMs are critical for the stability of ZIP13 protein, and compounds that inhibit protein degradation are potential therapeutics for SCD-EDS. Further exploration of the pathogenic mechanism of SCD-EDS will reveal new avenues for clinical interventions.

## Materials and Methods

### Cell culture and compounds

293T, HeLa, HT1080, and the human dermal fibroblast (Lonza) were maintained in DMEM+GlutaMAX medium (Gibco) with 10% FBS and antibiotics at 37°C. To construct stable cell lines, plasmids were transfected using Lipofectamine 2000 (Invitrogen), and cells were selected with 100 μg/mL HygroGold (Invivogen) for 293T cells and 10–50 μg/mL blasticidin (Invivogen) for HeLa cells. To monitor the amount of transfected plasmid, the cDNAs of ZIP13 and its mutants were subcloned into pMX-IRES-hCD8 (Yamasaki *et al*, 2006). Bafilomycin (Sigma), MG132 (Sigma), lactacystin (Enzo Life Sciences), PYR-41 (Sigma), DBeQ (Sigma), bortezomib (Cell Signaling), and cycloheximide (Sigma) were dissolved in DMSO.

### Plasmid constructs

FLAG-tagged ZIP13 and V5-tagged ZIP13 were constructed as previously described (Fukada *et al*, 2008; Bin *et al*, 2011). Plasmids used for the ubiquitination analysis were kind gifts from Drs. Takashi Tanaka and Chin Ha Jung. The plasmid encoding a dominant-negative form of VCP (E305Q/E578Q) (Shirogane *et al*, 1999) was reconstructed into p3xFLAG-Myc-CMV-26 (Sigma). The various G64 mutants were constructed using the EZchange™ Site-directed Mutagenesis kit (Enzynomics) with designated primers (Supplementary Table S1) as described by the manufacturer. The reporter vector pGL4.12-MT-264/+42 contained the mouse *MT*-*1* promoter was a gift from Dr. Tomoki Kimura (Kimura *et al*, 2008).

### Western blotting analysis

Cells were collected in 1% NP-40 containing 0.05 M Tris–HCl, pH 7.5, 0.15 M NaCl, and 0.01 M MgCl_2_. After centrifugation at 15,000 × *g* for 5 min, the supernatant was collected and analyzed as the soluble fraction. The pellet was re-suspended in 1% SDS containing 0.05 M Tris–HCl, pH 7.5, 0.15 M NaCl, and 0.01 M MgCl_2_ and analyzed as the insoluble fraction. Those fractions were boiled for 5 min in SDS–PAGE sample buffer containing 0.125 M Tris–HCl, pH 6.8, 20% glycerol, 4% SDS, 10% 2-mercaptoethanol, and 0.004% bromophenol blue and loaded onto a 5–20% or 10–20% polyacrylamide gradient gel. The ER stress antibody sampler kit was obtained from Cell Signaling Technology. Blue native-PAGE was performed as previously described (Bin *et al*, 2011). Anti-V5 (Invitrogen), anti-tubulin (Santa Cruz), anti-ubiquitinated proteins (Biomol), anti-FLAG (Sigma), and anti-VCP (Abcam) antibodies, and an anti-ER stress antibody sampler kit (Cell Signaling) were used for protein detection.

### Quantitative Real-time PCR

cDNA was synthesized using ReverTra Ace (Toyobo). The mRNA levels of ZIP13 were analyzed as previously reported (Bin *et al*, 2011). The mRNA levels of *CHOP* and *BIP* were analyzed using the TaqMan® Gene Expression Assay following the manufacturer's instructions (Applied Biosystems).

### Generation of anti-ZIP13 antibodies

The ab-A1 and ab-A2 anti-ZIP13 antibodies were generated in rabbits against synthetic peptides corresponding to amino acids 23–35 of human ZIP13 for ab-A1, and 184–201 of mouse ZIP13 for ab-A2 (Fukada *et al*, 2008). The monoclonal antibody 35B11 was produced using the method of Hino and others (Hino *et al*, 2012, 2013). Briefly, purified ZIP13 (Bin *et al*, 2011) was reconstituted into phospholipid vesicles consisting of egg L-α-phosphatidylcholine and Lipid A (Sigma) as an adjuvant. The antibodies were screened by ELISA and dot blot analyses, as described previously (Hino *et al*, 2013).

### Biacore

The binding affinity of 35B11 (IgG2a) for ZIP13 was tested by SPR spectroscopy using a Biacore T200 analyzer (GE Healthcare). A monoclonal anti-mouse Fcγ fragment-specific antibody was immobilized on a sensor chip (CM5), and the culture media after hybridoma cell cultivation were then loaded. Antibodies in the supernatant were tightly trapped by the anti-Fc antibody. The running buffer was 0.02 M HEPES (pH 7.4), 0.15 M NaCl, and 0.04% dodecyl-β-D-maltopyranoside (DDM). Purified ZIP13 protein in 0.04% DDM was then passed over the surface. Analyte concentrations were calculated using the absorbance at 280 nm with the theoretical extinction coefficients.

### Confocal microscopy

Cells were seeded onto glass coverslips in 35-mm glass dishes (Iwaki) overnight and were treated with or without 10 μM MG132 for 6 h. The cells were then fixed with 4% paraformaldehyde in PBS. For immunostaining, the cells were made permeable with BD Perm/Wash buffer containing antibodies and 1% BSA. Fluorescence was detected with an inverted spectral Confocal Scanning system, TCS SP2 AOBS (Leica), with an oil immersion 63× objective. Images were processed with Adobe Photoshop CS3 version 10.0. DAPI (Molecular Probes), anti-V5 antibody (Invitrogen), anti-GM130 antibody (clone35, BD Transduction Laboratories), and Alexa Fluor®635 phalloidin (Molecular Probes) were used to visualize nuclei, ZIP13, Golgi, and actin, respectively. Alexa Fluor®546 goat anti-mouse IgG F(ab')^2^ fragment was used for the secondary staining of GM130.

### Flow cytometric analysis

Cells were fixed and permeabilized with cytofix/cytoperm reagent (BD Biosciences) for 15 min at room temperature. After washing with Perm/Wash buffer, the cells were blocked with 0.5% BSA containing Perm/Wash buffer for 30 min at room temperature. The cells were then stained with 20 μg/ml anti-ZIP13 antibody (clone 35B11) in 0.5% BSA containing perm/wash buffer for 1 h at room temperature, washed extensively with Perm/Wash buffer, and then further incubated with goat anti-mouse Alexa 488 (Molecular Probes) for 1 h at room temperature. After more extensive washing with Perm/Wash buffer, the cells were subjected to flow cytometric analysis.

### Immunoprecipitation and mass spectrometry

Cells were disrupted in 1% NP-40 lysis buffer containing 0.05 M Tris–HCl, pH 7.5, 0.15 M NaCl, and 0.01 M MgCl_2_, and the cell debris was removed by centrifugation at 15,000 × *g* for 5 min. After incubation with an anti-V5 or anti-FLAG antibody for 3–4 h, the immune complexes were pulled down with protein G (GE Healthcare) for 2–3 h and then washed with 0.05% NP-40 lysis buffer. The complexes were dissociated in 1% SDS–PAGE sample buffer and subjected to SDS–PAGE and silver staining. Single bands were cut out and analyzed by mass spectrometry, and VCP (NP_009057.1) was identified.

### Ni-NTA purification

For Ni-NTA purification, cells were harvested into a denaturing lysis buffer (0.05 M Tris–HCl and 6 M GuHCl, adjusted to pH 8.0 using NaOH). The cell debris was disrupted by sonication, and Ni-NTA agarose was added. The mixture was then incubated for over 2 h. The Ni-NTA agarose was washed with 0.05 M Tris–HCl and 8 M urea, pH 6.3, and the proteins were eluted into 0.05 M Tris–HCl and 8 M urea, pH 4.5.

### siRNA transfection

Cells were transiently transfected with 100 pM siRNA (Genolution) using Lipofectamine RNAimax (Invitrogen), according to the manufacturer's instructions. VCP-targeting siRNAs were constructed using the human VCP mRNA sequence at nucleotides 599–619 (TGTAGGGTATGATGACATTG) or 480–500 (TAACCTTCGTGTACGCCTA). PA28-targeting siRNAs were constructed using the published human PA28 mRNA sequence (GAAUCAAUAUGUCACUCUAUU) or (UCUGAAGGAACCAAUCUUAUU) (Chen *et al*, 2007).

### Measurement of Zn level

Zn fluorescence staining was performed with slight modification (Taniguchi *et al*, 2013). 293T cells were treated with 10 nM bortezomib for 6 h. Afterwards, they were incubated with 1 μM FluoZin-3 for 30 min, and then with 10 μM Zn pyrithione for 10 min. The cells were washed with PBS and fixed with 4% paraformaldehyde in PBS. Fluorescence was detected with an inverted fluorescence imaging system, EVOS f1 (AMG). To quantify the cellular Zn level, 1 × 10^6^–10^7^ cells were subjected to a modified acid deproteinizing method (Nomoto, 1987) and then analyzed by inductively coupled plasma-atomic emission spectrometry (ICP-AES).

### Patient cells

Written informed consents were obtained from the subjects. The study was approved by ethics committees of participating institutions.

### Statistical analysis

The two-tailed Student's *t*-test was used to analyze the difference between two groups.

**The paper explained**

**Problem**

The spondylocheirodysplastic form of Ehlers-Danlos syndrome (SCD-EDS, OMIM 612350), a genetic disorder of connective tissues, bones, and teeth, is related to an imbalance in the cellular handling of zinc caused by mutation in the zinc transporter ZIP13; however, the pathogenic mechanism of the mutation is poorly understood.

**Results**

We found that pathogenic ZIP13 proteins are degraded by the VCP-linked ubiquitin proteasome pathway. Interrupting this pathway restored the ZIP13 expression levels, resulting in improvement of the intracellular Zn homeostasis.

**Impact**

Our data revealed the pathogenic mechanism of mutant ZIP13 proteins and lend credence to the therapeutic potential of inhibitors for proteasome-dependent pathways. Further studies may lead to new therapeutic intervention strategies for SCD-EDS.

## Supporting Information

for this article is available online: http://embomolmed.embopress.org
