# Supplementary material for: Molecular pathogenesis of Spondylocheirodysplastic Ehlers-Danlos syndrome caused by mutant ZIP13 proteins
Source: EMBO Mol Med. 2014 Jul 9;6(8):1028–42. doi: 10.15252/emmm.201303809 (PMC4154131; doi:10.15252/emmm.201303809)
Supplement: Supplementary file 1 [file emmm0006-1028-sd1.pdf]

Supplementary Information

Molecular pathogenesis of Spondylocheirodysplastic Ehlers-Danlos syndrome caused by mutant ZIP13 proteins

Bum-Ho Bin, Shintaro Hojyo, Toshiaki Hosaka, Jinhyuk Bhin, Hiroki Kano, Tomohiro Miyai, Mariko Ikeda, Tomomi Kimura-Someya, Mikako Shirouzu, Eun-Gyung Cho, Kazuhisa Fukue, Taiho Kambe, Wakana Ohashi, Kyu-Han Kim, Juyeon Seo, Dong-Hwa Choi, Yeon-Ju Nam, Daehee Hwang, Ayako Fukunaka, Yoshio Fujitani, Shigeyuki Yokoyama, Andrea Superti-Furga, Shiro Ikegawa, Tae Ryong Lee and Toshiyuki Fukada

Table of contents:

TABLE:

Supplementary Table 1.
Primers used for the construction of G64 mutants……………………………………..………………2

FIGURES:

Supplementary Figure 1.
Intracellular Zn level in wild-type or mutant ZIP13 expressing cell lines. 3

Supplementary Figure 2.
Decreased protein levels of the SCD-EDS pathogenic mutants of ZIP13 in stably expressing HeLa cells. ……….4

Supplementary Figure 3.
Increased dimers of G64D mutant protein in the presence of proteosome inhibitor MG132……..…...5

Supplementary Figure 4.
Equivalent *ZIP13* mRNA expression levels in cells transiently expressing wild-type ZIP13 (WT-V5), G64 mutants, and ΔFLA mutant (ΔFLA-V5). …………………………………………………….………6

Supplementary Figure 5.
The 20S proteasome is not significantly involved in the degradation of SCD-EDS pathogenic ZIP13 mutants. ….7

Supplementary Figure 6.
Degradation of the SCD-EDS pathogenic ZIP13 mutants involves ubiquitination. ……………………..8

Supplementary Figure 7.
Intracellular flow cytometric analysis for exogenous ZIP13 expression. …………………………………9

Supplementary Figure 8.
Bortezomib restored the mutant ZIP13 proteins and the intracellular Zn homeostasis. ……………….10

Supplementary Figure 9.
MG132 restored the intracellular Zn homeostasis. ………………………………………………………...11

Supplementary Figure 10.
HSP90 inhibitor treatment restores the G64D mutant ZIP13 protein level. ……………………………..12

Supplementary Figure 11.
Expression levels of ER stress responsive genes and proteins. …………………………………………13

REFERENCE…………………………………………………………………………………………...14

TABLE:

Supplementary Table 1. Primers used for the construction of G64 mutants

Mutant	Sense primer	Anti-sense primer
G64A	5'-GCTTCCCTCATGGTGGGGCTCAGTG	5'-CAGGAGGGAGCAGATCCAGGTGTC
G64C	5'-TGTTCCCTCATGGTGGGGCTCAGTG	5'-CAGGAGGGAGCAGATCCAGGTGTC
G64S	5'-TCTTCCCTCATGGTGGGGCTCAGTG	5'-CAGGAGGGAGCAGATCCAGGTGTC
G64I	5'-ATTTCCCTCATGGTGGGGCTCAGTG	5'-CAGGAGGGAGCAGATCCAGGTGTC
G64L	5'-CTTTCCCTCATGGTGGGGCTCAGTG	5'-CAGGAGGGAGCAGATCCAGGTGTC
G64E	5'-GAGTCCCTCATGGTGGGGCTCAG	5'-CAGGAGGGAGCAGATCCAGGTGTC
G64R	5'-AGATCCCTCATGGTGGGGCTCAG	5'-CAGGAGGGAGCAGATCCAGGTGTC
G64N	5'-AACTCCCTCATGGTGGGGCTCAGTGG	5'-CAGGAGGGAGCAGATCCAGGTGTC
G64Q	5'-CAGTCCCTCATGGTGGGGCTCAGTGG	5'-CAGGAGGGAGCAGATCCAGGTGTC
*Mutated sequences are underlined		

FIGURES:

Supplementary Figure 1

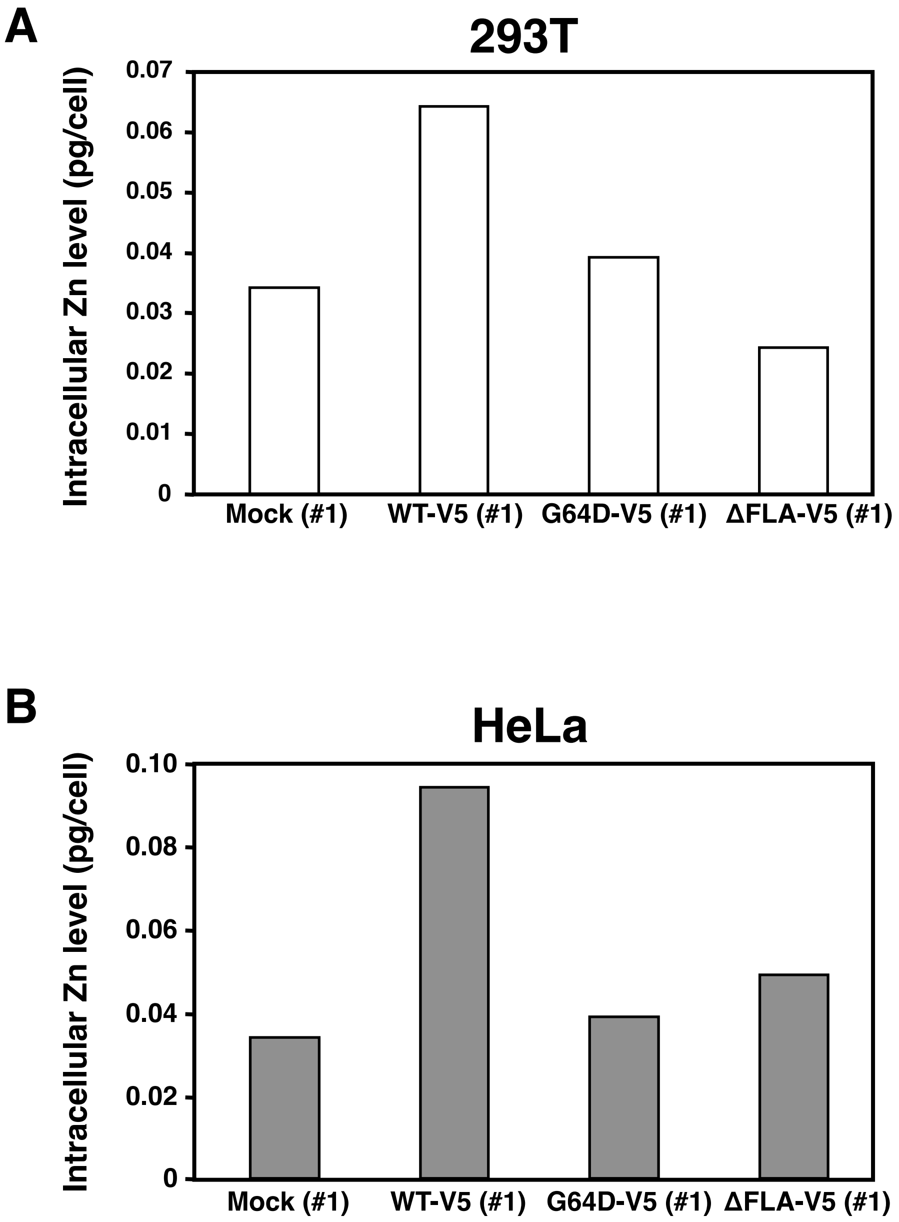

Fig S1. Intracellular Zn level in wild-type or mutant ZIP13 expressing cell lines.
Cellular Zn level was measured by ICP-AES in representative **(A)** 293T **(Fig 5)** and **(B)** HeLa clones **(Fig E2)** stably expressing Mock, wild-type (WT-V5), G64D mutant (G64D-V5), or ΔFLA mutant (ΔFLA-V5) vector.

Supplementary Figure 2

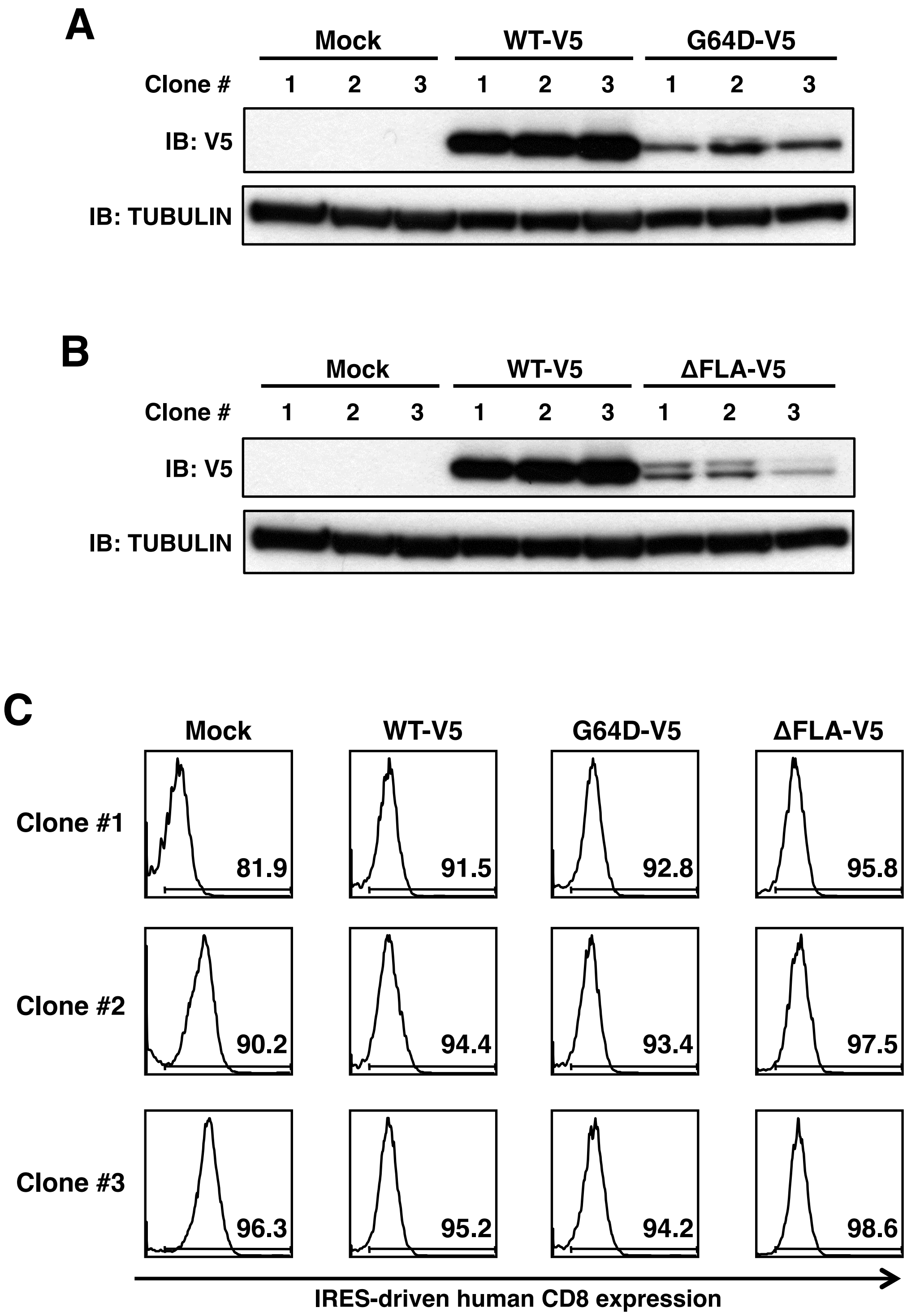

Fig S2. Decreased protein levels of the SCD-EDS pathogenic mutants of ZIP13 in stably expressing HeLa cells.

A. Protein levels of the G64D mutant ZIP13. Cell lysates of three representative HeLa clones stably expressing C-terminally V5 epitope-tagged WT-V5 or G64D-V5 ZIP13, were analyzed by western blotting using an anti-V5 antibody.

B. Protein levels of the ΔFLA mutant ZIP13. Cell lysates of three representative HeLa clones stably expressing WT-V5 or ΔFLA-V5 were analyzed by western blotting using an anti-V5 antibody.

C. The hCD8 expression level as an indicator of the amount of transfected plasmid DNA (pMX-WT-IRES-hCD8, pMX-G64D-IRES-hCD8, or pMX-ΔFLA-IRES-hCD8). Three representative HeLa clones stably expressing WT-V5, G64D-V5, or ΔFLA-V5, were analyzed by flow cytometry using an APC-conjugated anti-hCD8 antibody. Histograms were gated on hCD8-positive cells.

Supplementary Figure 3

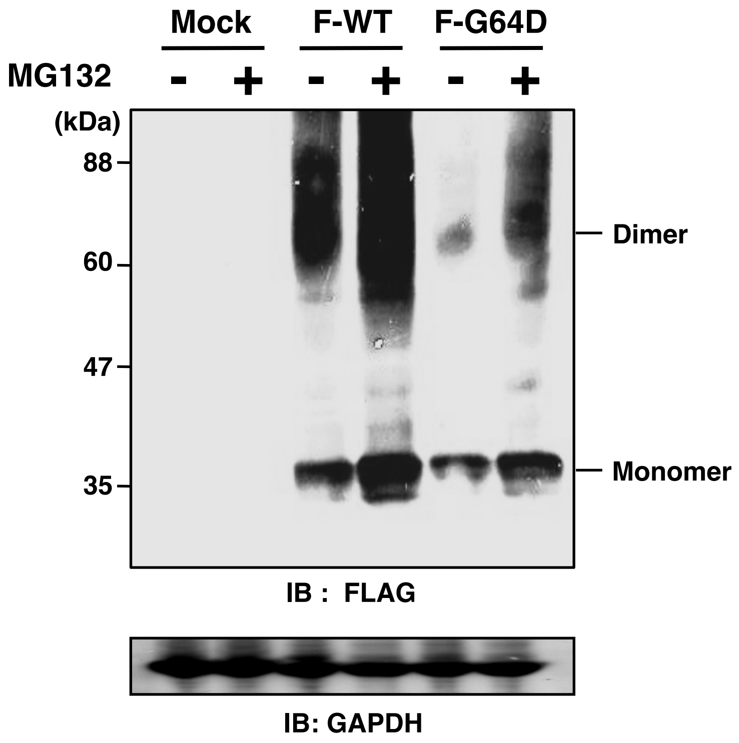

Fig S3. Increased dimers of G64D mutant protein in the presence of proteosome inhibitor MG132. The dimer formation of ZIP13 was analyzed by western blotting under non-reducing conditions using the lysates of HT1080 cells expressing N-terminally 3xFLAG-tagged wild-type (F-WT) and G64D mutant (F-G64D) ZIP13 (Bin et al, 2011).

Supplementary Figure 4

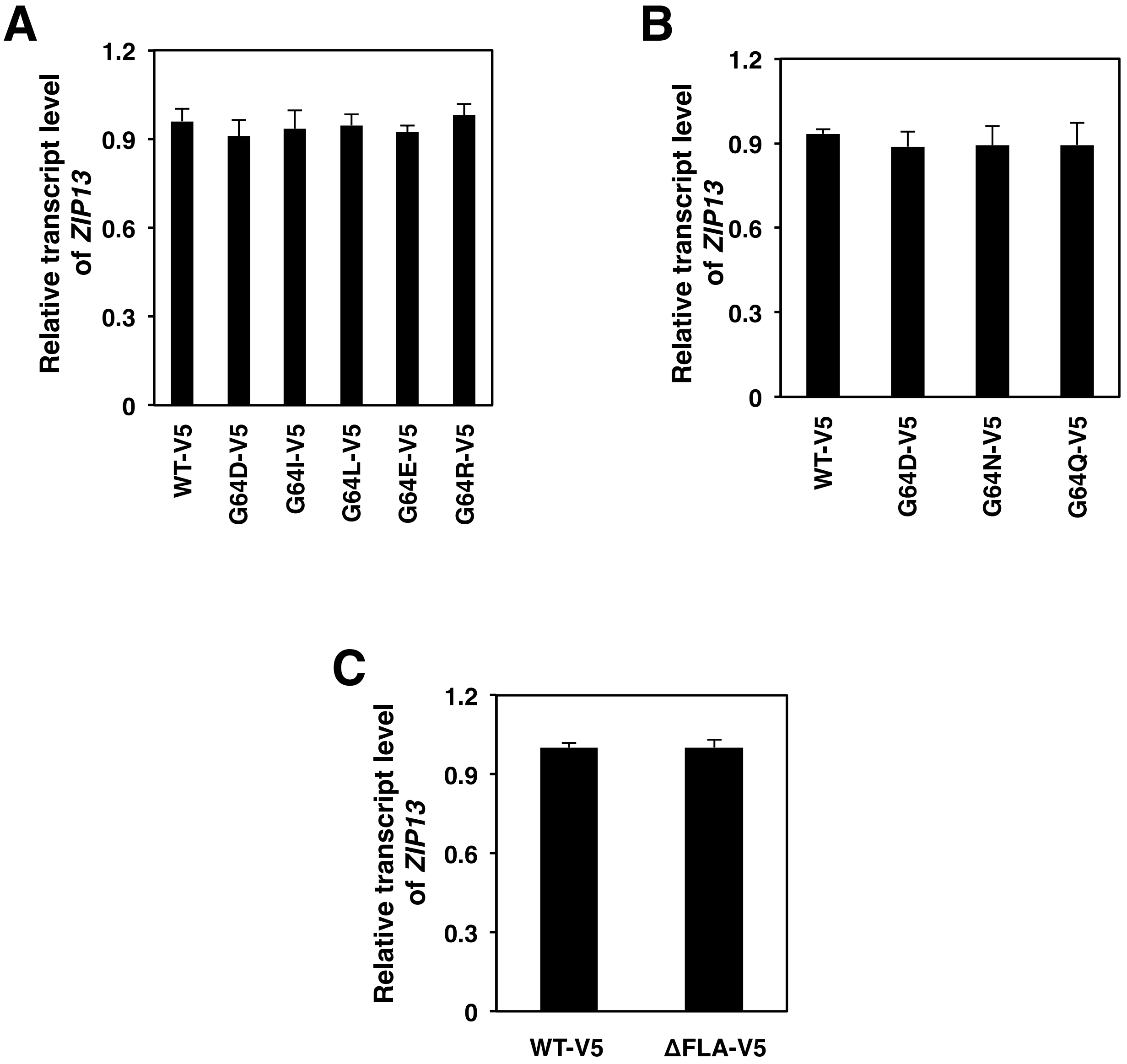

Fig S4. Equivalent *ZIP13* mRNA expression levels in cells transiently expressing wild-type ZIP13 (WT-V5), G64 mutants, and ΔFLA mutant (ΔFLA-V5).

A. The transcript levels of mutant constructs encoding ZIP13-V5 with various amino acids at position 64 were comparable to that of wild type.

B. The transcript levels of the mutant constructs encoding G64N-V5 and G64Q-V5 were comparable to that of wild type.

C. The transcript level of the mutant construct encoding ΔFLA-V5 was comparable to that of wild type.

Supplementary Figure 5

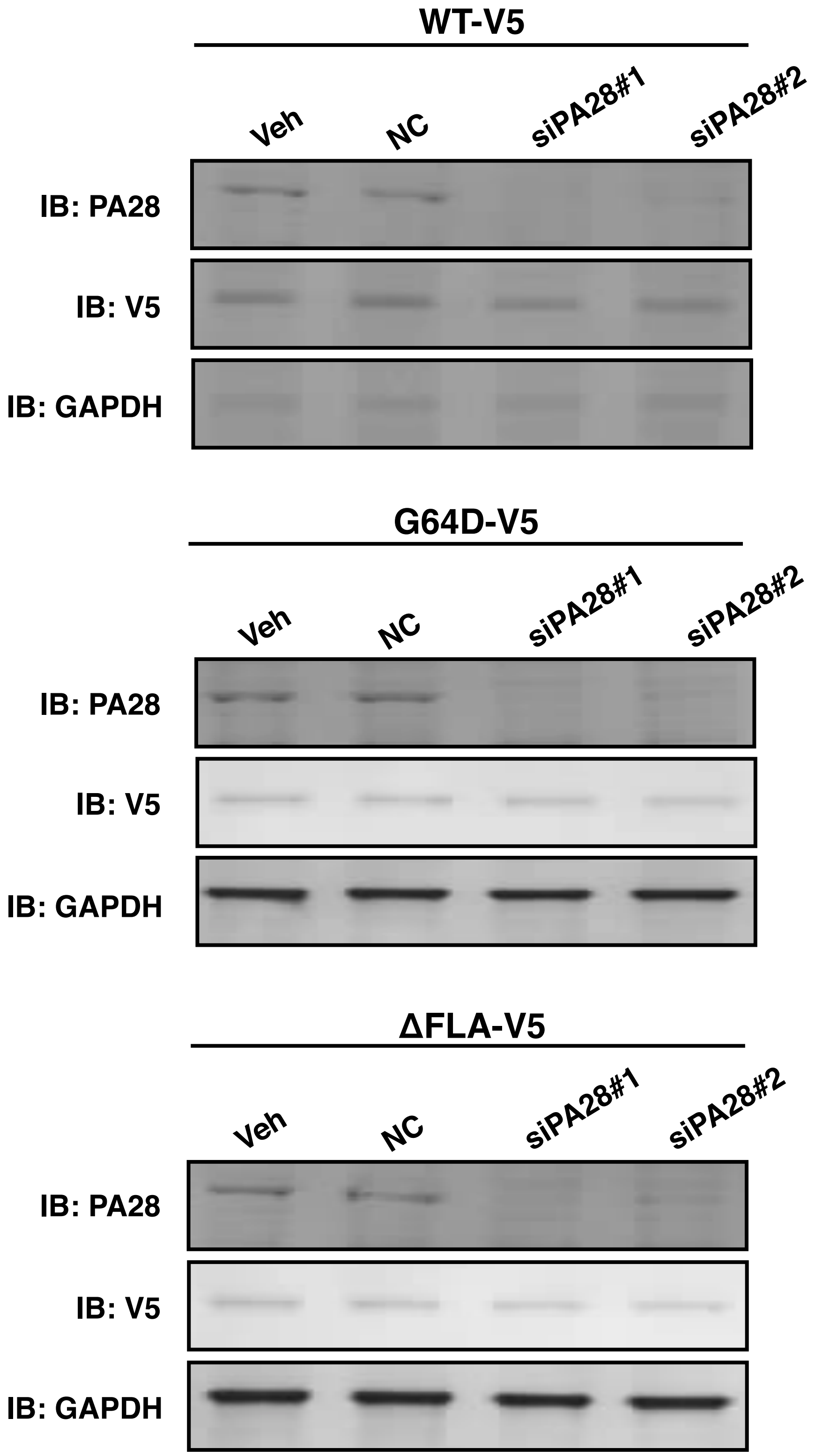

Fig S5. The 20S proteasome is not significantly involved in the degradation of SCD-EDS pathogenic ZIP13 mutants.
The siRNA targeting PA28, which induces the 20S proteasome, did not affect the protein expression of C-terminally V5 epitope-tagged WT-V5, G64D-V5, or ΔFLA-V5 ZIP13 in 293T cells. The siRNAs were transfected into 293T cells stably expressing each type of ZIP13 protein. Seventy-two hours post-transfection, the cells were harvested and subjected to western blotting using an anti-PA28 or anti-V5 antibody.

Supplementary Figure 6

A

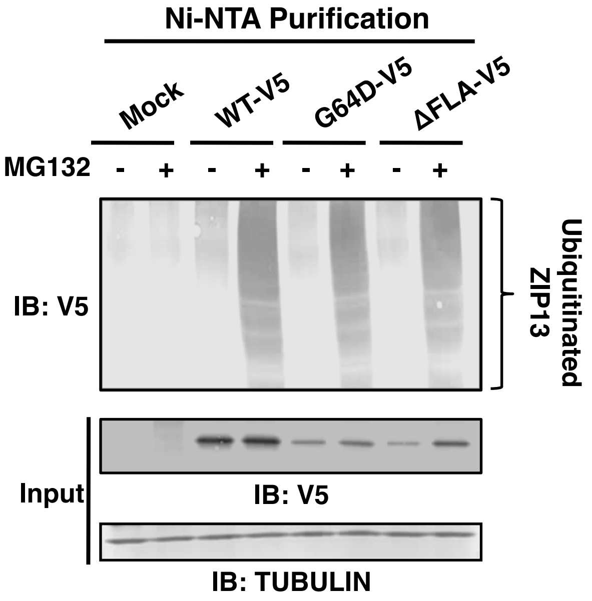

B

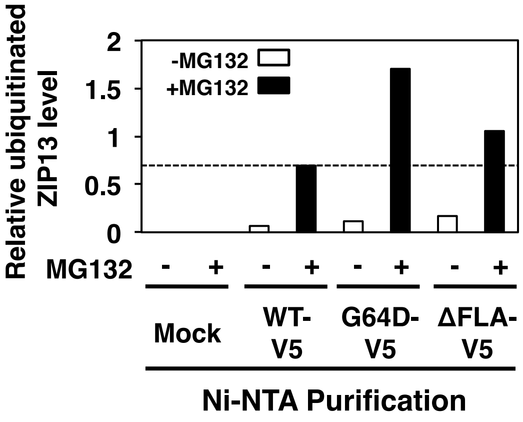

Fig S6. Degradation of the SCD-EDS pathogenic ZIP13 mutants involves ubiquitination.
A. Detection of ubiquitinated ZIP13 proteins. 293T cells co-expressing 6x histidine-tagged ubiquitin and WT-V5, G64D-V5, or ΔFLA-V5 were treated with 10 μM MG132 for 6 h, lysed with denaturing buffer, and purified by Ni-NTA agarose. The ZIP13 proteins in the purified samples were then subjected to western blotting. Ubiquitinated ZIP13 proteins were detected using an anti-V5 antibody.
B. The ubiquitinated/normal protein ratio for the wild-type and mutant ZIP13 proteins were shown.

Supplementary Figure 7

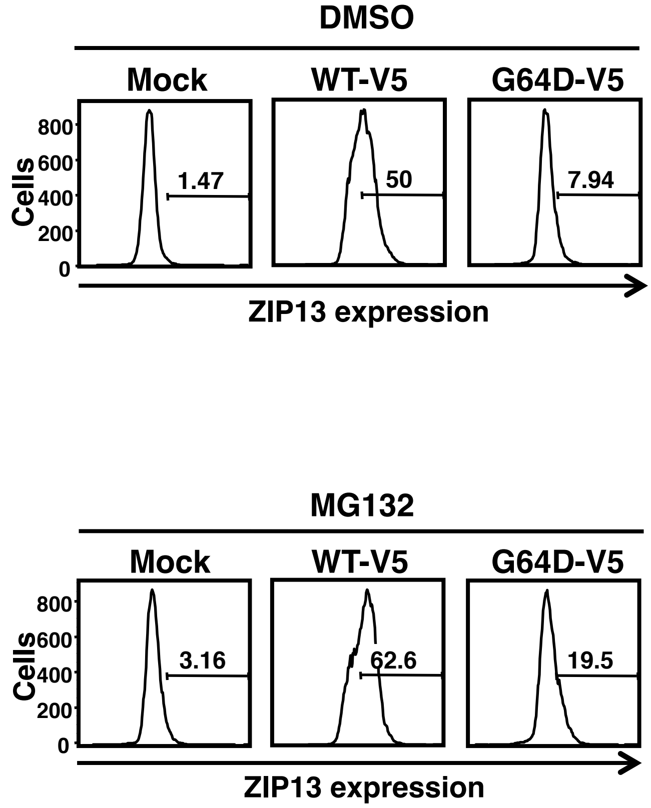

Fig S7. Intracellular flow cytometric analysis for exogenous ZIP13 expression.
HeLa stable clones expressing WT-V5 or G64D-V5 were treated with DMSO or 10 μM MG132. After fixation and permeabilization, the cells were stained with the monoclonal antibody 35B11, followed by goat-anti-mouse-Alexa 488.

Supplementary Figure 8

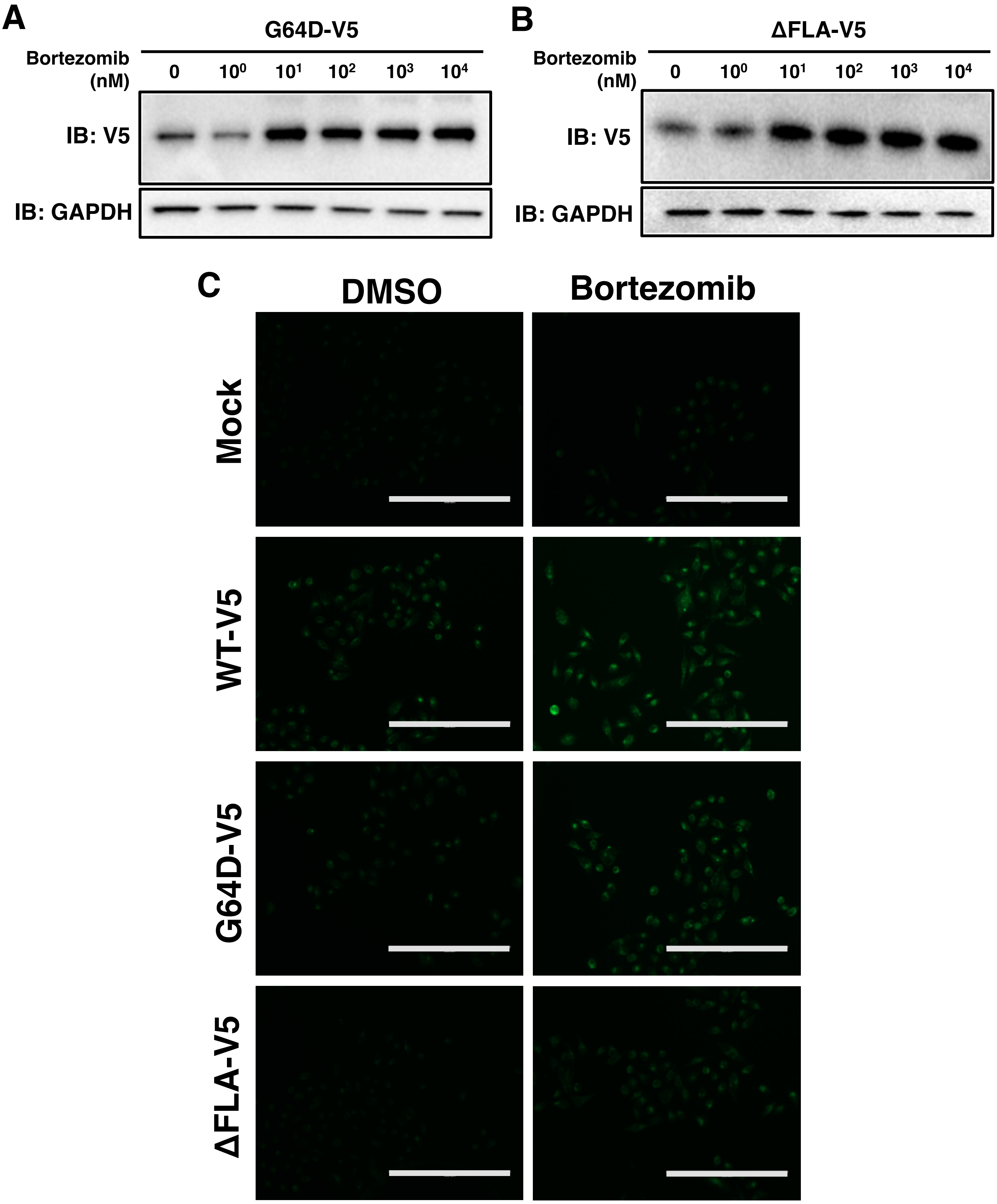

Fig S8. Bortezomib restored the mutant ZIP13 proteins and the intracellular Zn homeostasis.
A. 293T cells stably expressing G64D-V5 were treated with Bortezomib at the indicated concentrations, followed by western blotting using an anti-V5 antibody.
B. 293T cells stably expressing ΔFLA-V5 were treated with Bortezomib at the indicated concentrations, followed by western blotting using an anti-V5 antibody.
C. HeLa cells stably expressing WT-V5, G64D-V5, or ΔFLA-V5, were treated with 10 nM Bortezomib for 6 h. The intracellular Zn level was monitored by Fluozin-3 in combination with Zn pyrithione treatment. Bars, 200 μm.

Supplementary Figure 9

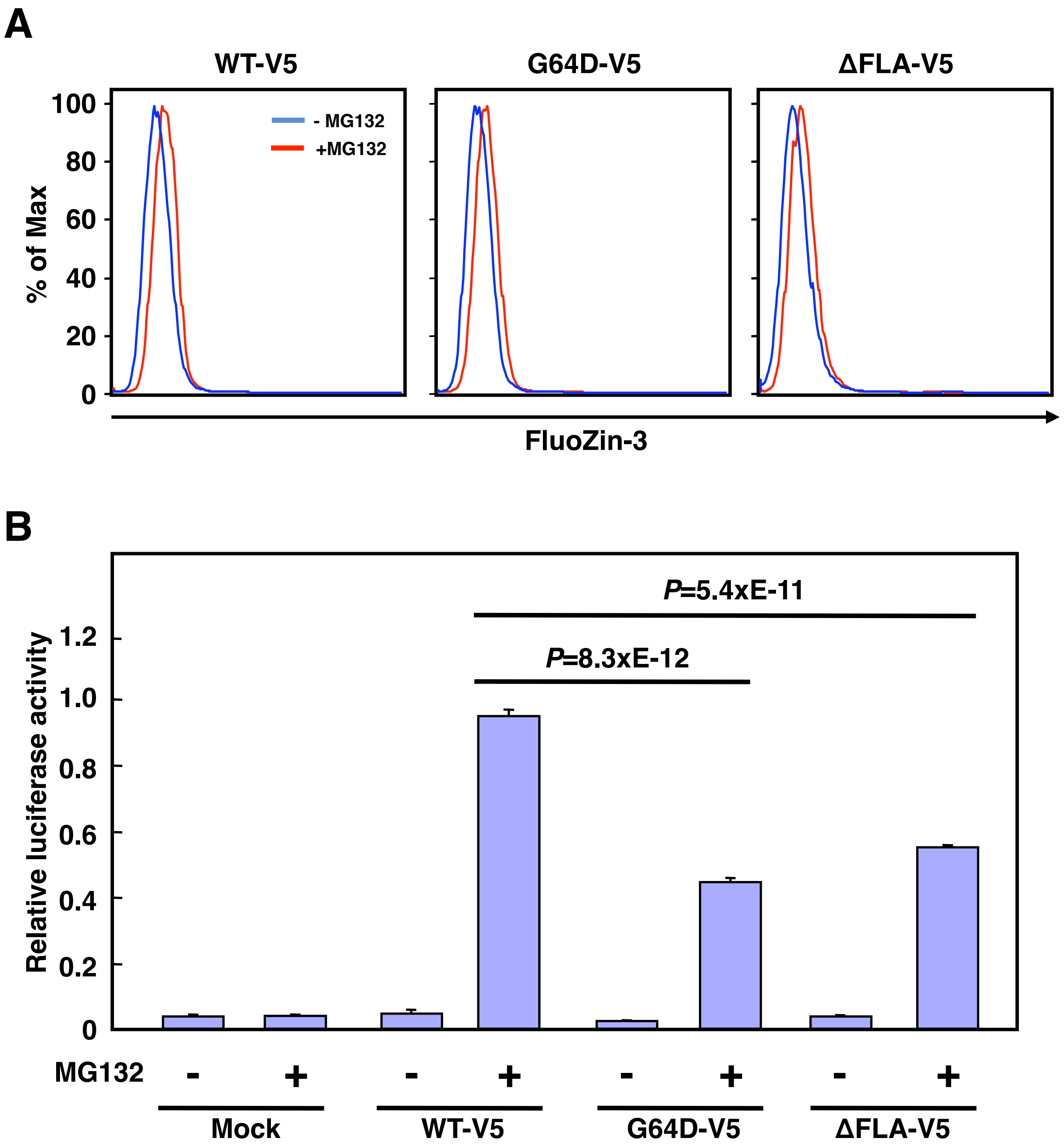

Fig S9. MG132 restored the intracellular Zn homeostasis.
A. HeLa cells stably expressing indicated expression plasmids were treated with DMSO (blue line) or 10 μM MG132 (red line) for 6 h, followed by incubation with 0.1 μM of FluoZin-3 and 7AAD. 7AAD-negative populations were subjected to flow cytometric analysis.
B. HeLa cells stably expressing indicated expression plasmids were transiently transfected with *MT1* promoter reporter and phRL-TK plasmids, followed by incubation of 10 μM MG132 for 6 h and analyzed the luciferase activity. Data are shown as mean ± s.e.m. (Student's t-test).

Supplementary Figure 10

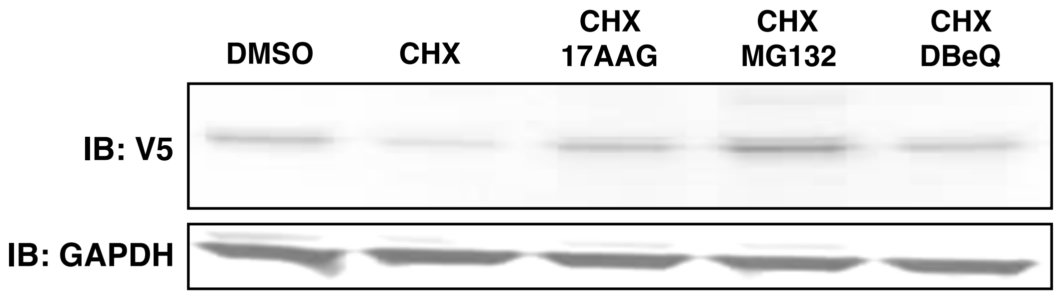

Fig S10. HSP90 inhibitor treatment restores the G64D mutant ZIP13 protein level.
Treatment with an HSP90 inhibitor, 17-(Allylamino)-17-demethoxygeldanamycin (17AAG), restored the protein level of G64D-V5 in the presence of CHX. 293T cells stably expressing G64D-V5 were treated with 10 μM 17AAG, 10 μM DBeQ, or 10 μM MG132 in the presence of CHX for 6 h. Total cell lysates were subjected to western blotting using an anti-V5 antibody.

Supplementary Figure 11

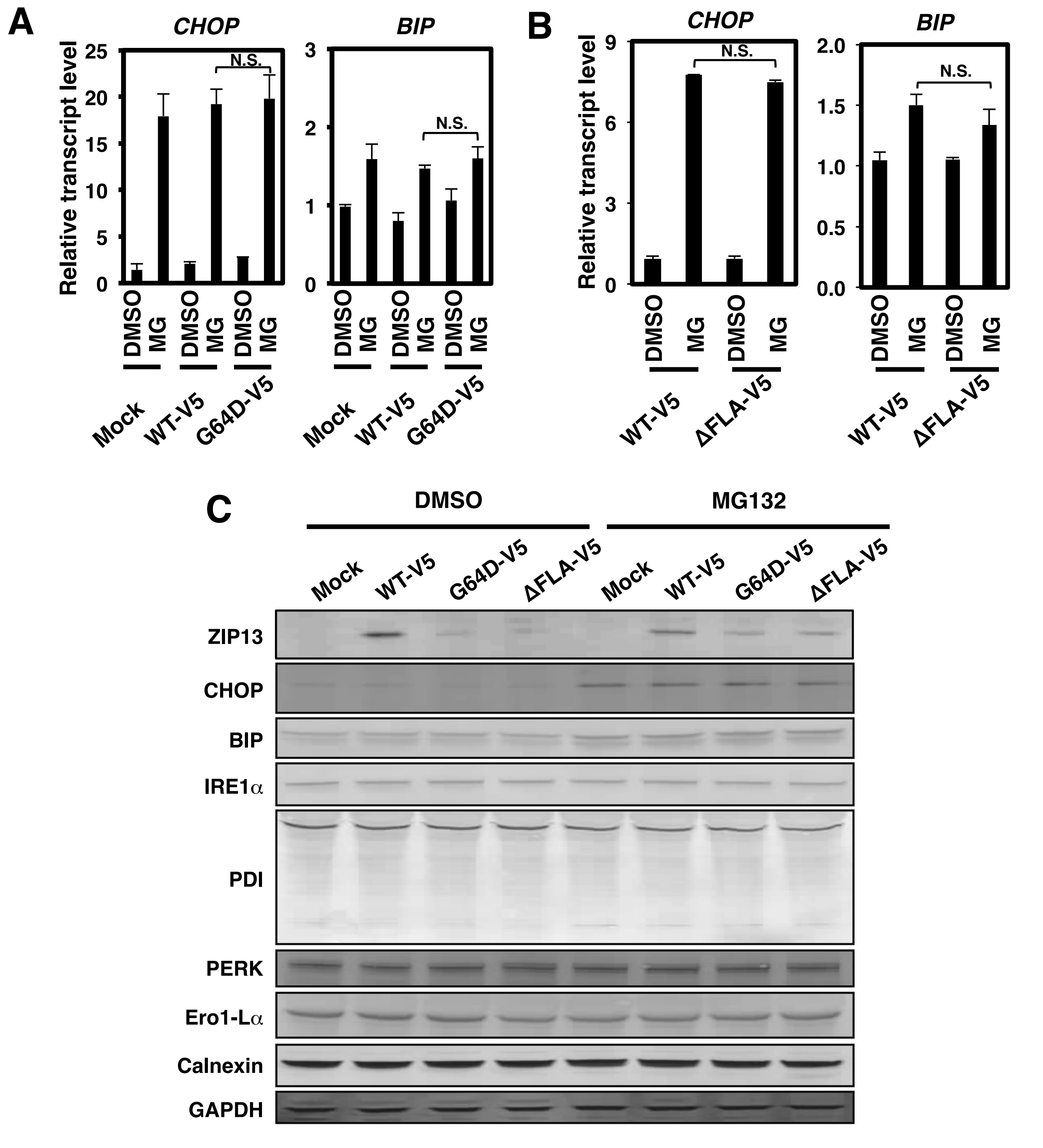

Fig S11. Expression levels of ER stress responsive genes and proteins.

A. The mRNA expression levels of *CHOP* and *BIP* in 293T cells expressing WT-V5 or G64D-V5. Cells were transfected with expression plasmids, and then treated with 0.5 μM MG132 for 6 h. The mRNA expression levels of *CHOP* and *BIP* were analyzed by RT-qPCR. Data are representative of three experiments, and shown as mean ± s.e.m. (Student's t-test). N.S.: not significant, MG: MG-132

B. The mRNA expression levels of *CHOP* and *BIP* in 293T cells expressing WT-V5 or ΔFLA-V5. RT-qPCR analysis for *CHOP* and *BIP* was performed as described in **A**. Data are representative of three experiments, and shown as mean ± s.e.m. (Student's t-test). N.S.: not significant; MG: MG-132

C. Protein expression levels of CHOP, BIP, IRE1α, PDI, PERK, Ero1-Lα, and calnexin in 293T cells expressing WT-V5, G64D-V5, or ΔFLA-V5. The cells were treated with 0.5 μM MG132 for 6 h. The cell lysate was subjected to SDS-PAGE and analyzed by western blotting using antibodies to the indicated proteins.

REFERENCE:

Bin BH, Fukada T, Hosaka T, Yamasaki S, Ohashi W, Hojyo S, Miyai T, Nishida K, Yokoyama S, Hirano T (2011) Biochemical characterization of human ZIP13 protein: a homo-dimerized zinc transporter involved in the Spondylocheiro dysplastic Ehlers-Danlos syndrome. ***J Biol Chem.*** 286**:** 40255-40265.
