# Supplementary material for: Molecular pathogenesis of Spondylocheirodysplastic Ehlers-Danlos syndrome caused by mutant ZIP13 proteins
Source: EMBO Mol Med. 2014 Jul 9;6(8):1028–42. doi: 10.15252/emmm.201303809 (PMC4154131; doi:10.15252/emmm.201303809)
Supplement: Supplementary file 2 [file emmm0006-1028-sd2.pdf]

Molecular pathogenic basis of Spondylocheirodysplastic Ehlers-Danlos syndrome caused by mutant ZIP13 proteins

Bum-Ho Bin, Shintaro Hojyo, Toshiaki Hosaka, Jinhyuk Bhin, Hiroki Kano, Tomohiro Miyai, Mariko Ikeda, Tomomi Kimura-Someya, Mikako Shirouzu, Eun-Gyung Cho, Kazuhisa Fukue, Taiho Kambe, Wakana Ohashi, Kyu-Han Kim, Juyeon Seo, Dong-Hwa Choi, Yeon-Ju Nam, Daehee Hwang, Ayako Fukunaka, Yoshio Fujitani, Shigeyuki Yokoyama, Andrea Superti-Furga, Shiro Ikegawa, Tae Ryong Lee and Toshiyuki Fukada

Corresponding author: Toshiyuki Fukada, RIKEN Center for Integrative Medical Sciences and Tae Ryong Lee Bioscience Research Institute

Review timeline:

Submission date:	29 December 2013
Editorial Decision:	05 January 2014
Appeal:	06 January 2014
Additional Editorial Correspondence:	07 January 2014
Additional Author Correspondence:	08 January 2014
Editorial Decision:	22 January 2014
Revision received:	24 April 2014
Editorial Decision:	12 May 2014
Revision received:	26 May 2014
Accepted:	27 May 2004

Transaction Report:

(Note: With the exception of the correction of typographical or spelling errors that could be a source of ambiguity, letters and reports are not edited. The original formatting of letters and referee reports may not be reflected in this compilation.)

Editor: Roberto Buccione

1st Editorial Decision 05 January 2014

Thank you for the submission of your manuscript "Molecular pathogenic basis of Spondylocheirodysplastic Ehlers-Danlos syndrome caused by mutant ZIP13 proteins" and apologies for not replying sooner due to the increasing number of submissions compounded with the Holiday season.

I have now had the opportunity to carefully read your paper and the related literature and I have also discussed it with my colleagues. I am afraid that we concluded that the manuscript is not well suited for publication in EMBO Molecular Medicine and have therefore decided not to proceed with peer review.

You find that the ZIP13-G64D protein is degraded by the valosin containing protein (VCP)-linked ubiquitin and proteasome-dependent pathway in a cell line in vitro and confirm that this mutation confers instability in cells from Spondylocheirodysplastic Ehlers-Danlos (SCD-EDS) patients and that ZIP13 expression was rescued with a proteasome inhibitor. You also find that the other

pathogenic mutant of ZIP13 (ZIP13deltaFLA) is similarly degraded in a cell line. We appreciate that the proteasome inhibitory drug Bortezomib restored expression of mutant ZIP13 proteins and cellular zinc homeostasis in vitro.

Although we acknowledge the potential interest of your findings, however, we feel that direct validation of the effects of bortezomib in the Zip13-KO model of SCD-EDS and possibly verification of ZIP13deltaFLA instability in SCD-EDS patient cells would be needed to strengthen the translational significance of your work.

If you can provide this experimental support in a future version, I would commit to sending your manuscript out for peer-review.

I am sorry that I could not bring better news this time.

Appeal 06 January 2014

First of all, I would like to express my heartfelt thanks to your efforts with careful reading and potential interests to our manuscript entitled "Molecular pathogenic basis of Spondylocheirodysplastic Ehlers-Danlos syndrome (SCD-EDS) caused by mutant ZIP13 proteins". Also, I would appreciate your important questions very much for further review process. I address your queries as below.

Q1: Direct validation of the effects of Bortezomib in the Zip13-KO model of SCD-EDS.
A1: I am afraid that it would not be an appropriate approach to ask the effects of Bortezomib in vivo, since Zip13-KO mouse is the ZIP13-"null" mouse. Therefore, to address this issue, we are currently trying to generate "knock-in" mouse which possesses G64D or delta mutation, as we mentioned in our manuscript (please see line 14 at page 9). These mice would be very helpful as SCD-EDS rodent models to evaluate the usefulness of Bortezomib prior to a clinical investigation, and to discover the other potential drugs as well. This issue will be addressed in the future study.

Q2: Possibly verification of ZIP13 delta instability in SCD-EDS patient cells.
A2: It will be certainly possible to pursue.

One thing I would like to emphasize is that this paper is the first demonstrating the molecular characteristics of pathogenic ZIP13 protein in detail, which is a very important step leading the translational research of the recently identified SCD-EDS caused by ZIP13 due to the fact that no possible treatments have been suggested as of now, so that we do believe our works have the relevance to look into the mechanisms of this intractable disease, in order to convert the basic knowledge described herein to the further clinical investigations in future, which will bring various benefits not only to the progress of medical science in general, but also into the hope of SCD-EDS patients in the world; I would assume that these contexts in our study will reasonably mesh with the policy of EMBO Molecular Medicine.

Therefore, your generous considerations to our manuscript must be appreciated very much.

If you have any other queries, please do not hesitate to get back to myself.

I am looking forward to reading the feedbacks from reviewers.

Additional Editorial Correspondence 07 January 2014

Thank you for your message.

You are of course right about the mouse model. The ZIP13 KO would certainly not be applicable to test Bortezomib! The lack of a pre-clinical validation in a mouse model remains a limitation of your study but we do confirm our interest and obviously understand that it will take time to generate the

knock-in model.

Although I am willing at this point to send out your manuscript for peer-review, it would be preferable if you could provide the verification of ZIP13 delta mutation instability in SCD-EDS patient cells.

Would you be willing/able to perform such experimentation within a short time frame?

Additional Author Correspondence 08 January 2014

I very much appreciate your prompt replay with the understanding of the important points of our studies.

Regarding the experiments using patient's cells, the experimental methods are simple, so all we can say is that we will do our best for them during the review and revision processes.

Since we first discovered both physiological roles of ZIP13 in mouse and human (Fukada et al 2008; already cited 80-times according to Google Scholar), this molecule has been paid more attention than ever, and at the same time, the elucidation of its patho-physiological roles in bone, tooth, ocular and connective tissues as well as in related metabolic diseases has gotten more competitive. Therefore, we have a particularly strong hope for the comments from peer-reviewers as soon as possible.

Once again, thank you very much.

2nd Editorial Decision 22 January 2014

Thank you for the submission of your manuscript to EMBO Molecular Medicine. We have now heard back from the three Reviewers whom we asked to evaluate your manuscript.
You will see that all three Reviewers are generally supportive of your work although a few concerns are expressed that require your action.

Reviewer 1 is concerned that wild-type and mutant ZIP13 show comparable stabilisation with the proteasome inhibitor, which would appear to conflict with the notion of the reduced stability of the mutant form. S/he suggests that half-life studies should be undertaken to address the issue. I wholeheartedly agree, especially since Reviewer 3 raises similar concerns. Furthermore, the whole issue requires some additional discussion, including with respect to VCP interaction. This Reviewer also provides additional comments for your consideration.

Reviewer 2 would like to see less indirect measurements of cytosolic Zn and suggests some avenues to pursue this. S/he also notes that the actual zinc transporter function of the mutant ZIP13 forms should be better addressed. This is indeed a relevant issue, which is also noted by Reviewer 3. This Reviewer also appears concerned that the cell models used are not fully appropriate in the context of EDS. Although I would suggest that you provide some degree of validation in alternative cell models if possible, I acknowledge that ERAD is so conserved as to make it unlikely that the process would be different in other cells. Reviewer 2 also raises additional points for you to act upon and suggests that some effort should be devoted to compacting the manuscript.

Reviewer 3 also notes the apparent discrepancy determined by the obvious ubiquitylation of wild-type ZIP13 and its association with VCP. As mentioned, Reviewer 1 has the same concern. It is thus of fundamental importance that the data are analysed carefully and discussed convincingly, with further experimentation where necessary. Reviewer 3 would like you to discuss the parallelism between the etiopathogenesis of EDS and cystic fibrosis and how this reflects on the therapeutic strategy proposed here and those in effect for cystic fibrosis. The Reviewer is also concerned with the insufficiently quantitative nature of the data in Figure 7c. Finally, s/he recommends working on English usage throughout the manuscript. I agree that while this issue has not been problematical, an

improved presentation would be quite beneficial.

While publication of the paper cannot be considered at this stage, we would be pleased to consider a suitably revised submission, with the understanding that the Reviewers' concerns must be fully addressed with additional experimental data where appropriate and that acceptance of the manuscript will entail a second round of review.

Please note that it is EMBO Molecular Medicine policy to allow a single round of revision only and that, therefore, acceptance or rejection of the manuscript will depend on the completeness of your responses included in the next, final version of the manuscript.

As you know, EMBO Molecular Medicine has a "scooping protection" policy, whereby similar findings that are published by others during review or revision are not a criterion for rejection. However, I do ask you to get in touch with us after three months if you have not completed your revision, to update us on the status. Please also contact us as soon as possible if similar work is published elsewhere.

I look forward to seeing a revised form of your manuscript as soon as possible.

***** Reviewer's comments *****

Referee #1 (Remarks):

In their paper entitled "Molecular pathogenic basis of Spondylocheirodysplastic Ehlers-Danlos syndrome caused by mutant ZIP13 proteins", Bin et. al. describe how two specific mutations in ZIP13 result in protein instability and degradation via the VCP-mediated ubiquitin dependent proteasome pathway. They provide a comprehensive and thorough analysis to provide convincing evidence supporting their hypothesis. In addition, they analyze primary samples from SCD-EDS patients, which provides further support for the physiological relevance and therapeutic implications of their conclusions. Overall the paper provides new insight into the molecular basis of SCD-EDS and potential avenues for successful therapeutic intervention. Specific comments and some minor grammatical corrections are detailed below.
1) Introduction, 2nd paragraph, recently characterized, not a recently characterized. Also need a comma after "as a new type of EDS,
2) Introduction, 2nd paragraph, patients show short stature (not statue).
3) Introduction, 2nd paragraph, "However, it remains unknown how..."
4) Introduction, final paragraph, "This is the first evidence demonstrating how these particular mutations..."
5) Pg 4. " in contrast, when the cells expressing mutant ZIP13 were treated similarly, the band-b was severely decreased while the band-A remained intact."
6) Pg 4. "...the V5-tagged mutant (G64D-V5) expression levels were lower."
7) Pg 5. In arguing that the loss of function is due mainly to decreased levels, it would be nice to show that dimerization of the mutant occurs/is enhanced in the presence of Mg132.
8) Fig. 3. The authors need to address why the wild type protein shows a comparable stabilization with Mg132. In other words, if G64D is less stable, then would expect to see greater stabilization with Mg132. This is confusing since later on it looks like the wt is much more stable. One way to address this directly is to determine the half-lives for the wt and mutant (eg using cyclohexamide). This is somewhat done in fig 4C. It appears that something more may be going on with G64D since its levels do not return to that of wt in the presence of Mg132. Likewise, I would expect to see more ubiquitinated G64D than wt, but the opposite occurs (fig 4b). In contrast, the deltaFLA appears more unstable and its levels return to those of wt (fig 5f). Since the authors have ruled out transcription I am wondering if there is also some translational control.
9) Pg 7. "...indicating that the VCP protein might preferentially interact with pathogenic ZIP13G64D."
10) Pg 8. The argument that VCP may recognize a conformational change caused by the mutation seems unlikely since the wild type is also ubiquitinated and also appears to be degraded by the proteasome.
11) The bortezomib results are a little weak, particularly with the deltaFLA mutant (fig 7AB). Why is this?

12) I do not find the model (fig 8) very convincing given the data. Why do the authors think the mutants insert in the membrane (at which point they would seem to be stabilized and/or difficult to direct the proteasome) rather than improperly folding (eg such that the typically hidden VCP binding domain is revealed) and hence targeted for the proteasome before reaching the membrane?

Referee #2 (Comments on Novelty/Model System):

All the in vitro experiments were performed in 293T cells or Hela cells, and the results are indeed convincing. However, SCD-EDS mainly involved connective tissues; therefore validation on cell lines derived from connective tissue may be more relevant to further clarify the relationship between ZIP13 mutations and the disease.

Referee #2 (Remarks):

The manuscript entitled "Molecular pathogenic basis of Spondylocheirodysplastic Ehlers-Danlos syndrome caused by mutant ZIP13 proteins" by Bin et al., demonstrated the molecular mechanism how ZIP13G64D and ZIP13 FLA mutations of SLC39A13 gene can lead to spondylocheirodysplastic form of the Ehlers-Danlos syndrome (SCD-EDS), and indicated that the disease results from the rapid degradation of mutant ZIP13 proteins via valosin containing protein (VCP)-linked ubiquitin and proteasome dependent pathway. This is very comprehensive study which elucidates the underlying mechanism of SCD-EDS, and investigated the biochemical process of ZIP13 protein degradation. The topic is interesting and may provide important information on the pathogenesis of SCD-EDS. The manuscript could be strengthened if the following questions were adequately addressed:

1. Novelty. It seems the role of ZIP13 in multiple cellular processes and diseases have been well established by the authors' group and other groups. The data included here in this manuscript represents incremental progress of ZIP13 and dysregulated zinc transport in EDS, although the data are convincing.
2. P4, L5, the metallothionein 1 gene as an indicator for the cytosolic Zn level is indirect. More direct evidence should be included such as AAS, ICPMS, or fluorescence detection.
3. P4, L9, the authors should show the effect of ZIP13G64D on Zn levels, which directly reflects the Zn homeostasis.
4. All the in vitro experiments were performed in 293T cells or Hela cells, and the results are indeed convincing. However, SCD-EDS mainly involved connective tissues; therefore validation on cell lines derived from connective tissue may be more relevant to further clarify the relationship between ZIP13 mutations and the disease.
5. Fig. 4C, the figure legend and the right panel is inconsistent (including DbeQ or not), statistics is also strongly suggested for this figure.
6. The result showed that the proteasome inhibitor can increase the expression level of ZIP13G64D and ZIP13 FLA, the two mutant proteins. The author should clarify whether these mutant proteins restore their normal zinc uptake function.
7. P5, L20, IRES need to be spelled out.
8. Several important studies were missing in the reference list on zinc transporters (ZIP13, ZIP4, ZIP6, etc) in various diseases. i.e. Potocki J Inorg Biochem 2013, Ou J Cell Biochem 2012, Zhang EMBO Mol Med 2013, Li PNAS 2007, Taylor Mol Med 2007, Hogstrand Biochem J 2013, Aydemir Gastroenterology 2012, Ryu PNAS 2011.
9. The manuscript is lengthy, suggest to shorten it especially the results section.

Referee #3 (Comments on Novelty/Model System):

With the exception of the data presented in Figure 7c, the data are technically sound and findings are often demonstrated using multiple approaches to cross-check.

While the concept of mutant transmembrane proteins being degraded by the proteasome via a VCP-

mediated process is not novel, the finding that mutant ZIP13 is degraded in this pathway in new, and it provides another disease link for VCP, a central protein to several vital cellular processes.

The model system chosen was human cell lines. This is appropriate given the authors' limited goal of demonstrating the molecular basis of a particular form of Ehlers Danlos Syndrome.

Referee #3 (Remarks):

Summary:

In the article by Bin et. al., entitled "Molecular pathogenic basis of Spondylocheirodysplastic Ehlers-Danlos syndrome caused by mutant ZIP13 proteins" the authors provided convincing primary and supporting evidence that two of the causative mutations of this form of Ehlers-Danlos syndrome, ZIP13 G64D and ZIP13 delta-FLA, result in a significantly shorter half-life for the ZIP13 protein. The authors demonstrated that this reduced half-life was due to ZIP13 ubiquitylation, followed by degradation by the proteasome (Figures 1 - 5), and mediated by the AAA ATPase, VCP (Figure 6). Finally, the authors provide data evaluating the ability of the FDA approved proteasome inhibitor, Velcade, to increase intracellular zinc levels in cells expressing mutant ZIP13.

Review:

The data provided for Figures 1-6 were technically sound, and were convincing in that often multiple experimental strategies were used in support of findings. The only pieces of data that appear contradictory to the authors thesis are the obvious ubiquitylation of wild-type ZIP13 and its association with VCP: figure 4B and figures 6A-6B, respectively. However, this apparent discrepancy can be readily explained by normal steady-state turnover of wild-type ZIP13, presumably occurring through the same/similar pathway. In addition, there is obviously more wild-type protein in these experiments, contributing to the appearance of increased ubiquitylation and VCP association to wild-type ZIP13. I would recommend that the authors address these data more thoroughly in their manuscript so that readers are not unnecessarily confused by the findings.

In figure 7 (more below), and in the discussion, the authors place a significant degree of attention upon the potential role of proteasome inhibitors, such as Velcade, as potential drug candidates to alleviate some of the symptoms of this Ehlers Danlos Syndrome. However, therapeutic interventions of diseases with similar molecular pathogenesis (e.g. cystic fibrosis), have not taken this approach (likely due to the severe side effects associated with prolonged proteasome inhibitor treatment in humans, such as peripheral neuropathy). Instead, molecular "potentiators" and "correctors" are in the clinic or in development (these drugs/drug candidates either allow the small fraction of mutant CFTR to function more effectively at the cell surface, or allow more mutant CFTR to reach the cell surface, respectively; an example potentiator is Kalydeco, for treatment of patients with the G551D CFTR mutation). The authors cite VX-809, which is in the "corrector" class. Given the obvious parallels of this study to CFTR mutations, I think it would be informative to the reader if the authors included additional detail in their discussion section to cover the therapeutic strategies found to be successful in treating cystic fibrosis, as well as some of the still experimental strategies (it is noted that VX-809 is included in their discussion).

The one set of data that were not as convincing as the other data presented in this manuscript was that of Figure 7c, where HeLa cells stably expressing WT, G64D, or delta-FLA were treated or not with Velcade and intracellular zinc levels were determined by qualitative microscopy. First, it appears that wild-type ZIP13 and G64D ZIP13 have identical increases in intensity (Zn concentration) over the mock transfection, with OR without proteasome inhibition; This finding does not correlate with decreased function of G64D ZIP13. Second, proteasome inhibition appears to increase zinc concentration in Mock transfected cells as well. Third (and of a lower concern), no comparison of zinc levels in a ZIP13 knockdown strains is made for our reference.

The major problem with the data in figure 7c is that they are not quantitative. I would recommend that the authors consider identifying a quantitative approach to determining intracellular zinc levels that would complement the microscopy experiments, or simply remove figure 7c from the manuscript (As mentioned above, it is this reviewer's opinion that the potential is small for current proteasome inhibitors like Velcade to be viable therapeutic candidates for treatment of diseases like

EDS). Figures 7A and 7B make the point that Velcade works similarly to research use only proteasome inhibitors.

Regarding the quality of the article's English language grammar, I would recommend that the authors have the manuscript further edited by an English language expert. While the number of typos did not prevent my understanding of their article in any way, clean-up would enhance the professional appearance.

1st Revision - authors' response 24 April 2014

I am very grateful to the editor and the reviewers for their constructive comments and suggestions; all three reviewers noted the value of our work. In particular, I thank Reviewers #1 and #2 for their highly supportive comments, which revealed an insightful understanding of zinc transporter biology. I also deeply appreciate the constructive comments from Reviewer #3, who shared valuable advice from clinical and pharmacological viewpoints.

Based on the comments and critiques of the editor and reviewers, we have revised our manuscript by addressing the reviewers' comments and making the manuscript more concise. These revisions include new results from additional experiments. The following three issues were commonly raised by the reviewers:

1: Ubiquitination and VCP association of wild-type ZIP13
2: Quantitative analysis of intracellular zinc
3: Additional cell models that better reflect the SCD-EDS pathogenesis

We have addressed these points as follows:

A1: We examined the ubiquitination and VCP association of the wild-type ZIP13, to determine the effects of normal steady-state turnover of the protein, as suggested by Reviewer #3.
A2: We performed ICP-AES and a flow cytometric analysis by zinc probe to measure the intracellular zinc level.
A3: We added analyses in a human dermal fibroblast (Fig 5H) and its cell line HT1080 (Fig E3).

In the present revised paper, therefore, we emphasize that this is the first demonstration of the importance of the physiological roles of the zinc transporter ZIP13 through its pathogenic consequences; the mutant ZIP13 proteins in SCD-EDS lose their function because they are degraded by the proteosome-dependent pathway in patients' cells. Because this paper reveals the molecular events underlying a newly identified human intractable disease, and suggests potential clinical applications, we feel strongly that it meets the criteria for *EMBO Molecular Medicine*. We believe it will appeal to a broad readership, and will hold particular interest for researchers interested in the molecular basis of disease.

Responses to reviewers' comments:

Reviewer #1:

We thank Reviewer #1 for the constructive comments, in which she/he noted the importance of our study of the mechanism of the pathogenic zinc transporter ZIP13 proteins. This reviewer mainly commented on the relevance of the ubiquitination and VCP association to wild-type ZIP13, quantitative analysis of the intracellular zinc level, and another cell model that reflects SCD-EDS. This reviewer also suggested the following corrections and improvements for descriptions in the manuscript.

Q1). Introduction, 2nd paragraph, recently characterized, not a recently characterized. Also need a comma after "as a new type of EDS,
Q2). Introduction, 2nd paragraph, patients show short stature (not statue).
Q3). Introduction, 2nd paragraph, "However, it remains unknown how..."
Q4). Introduction, final paragraph, "This is the first evidence demonstrating how these particular mutations..."

Q5). Pg 4. "in contrast, when the cells expressing mutant ZIP13 were treated similarly, the band-b was severely decreased while the band-A remained intact."
Q6). Pg 4. "...the V5-tagged mutant (G64D-V5) expression levels were lower."

A1-6: Many thanks for these notes. We rewrote these sentences as suggested prior to submitting the paper for English editing.

Q7). Pg 5. In arguing that the loss of function is due mainly to decreased levels, it would be nice to show that dimerization of the mutant occurs/is enhanced in the presence of Mg132.

A7: We appreciate this comment. We confirmed that the dimerization of the mutant protein was enhanced in the presence of MG132, by using the human fibroblast cell line HT1080, in the new Figure E3. HT1080 cells are fibroblast-related cells, and thus are useful as an additional cell model reflecting the disease.

Q8). Fig. 3. The authors need to address why the wild type protein shows a comparable stabilization with Mg132. In other words, if G64D is less stable, then would expect to see greater stabilization with Mg132. This is confusing since later on it looks like the wt is much more stable. One way to address this directly is to determine the half-lives for the wt and mutant (e.g. using cyclohexamide). This is somewhat done in fig 4C. It appears that something more may be going on with G64D since its levels do not return to that of wt in the presence of Mg132. Likewise, I would expect to see more ubiquitinated G64D than wt, but the opposite occurs (fig 4b).

A8: We thank the reviewer for these important comments. The G64D mutation led to its accumulation in the detergent-insoluble fraction, most likely due to changes in the properties of the ZIP13 protein (Figs 3A and G). To avoid presenting confusing results, we now show the ubiquitinated/non-ubiquitinated protein ratio (Figs 4B and E6), which demonstrates that the mutant proteins undergo greater ubiquitination than wild type, supporting the idea that the G64D protein is more unstable and easily ubiquitinated than the wild-type one. Since we totally agree with the suggestion raised by Reviewer #3 that "*this apparent discrepancy can be readily explained by normal steady-state turnover of wild-type ZIP13, presumably occurring through the same/similar pathway,*" we inserted the following sentences to clarify this point.
- line 14 on page 5
"While Balifomycin had no apparent effect on the protein expression patterns, MG132 preferentially increased the amount of WT-V5 and G64D-V5 protein in the NP40-detergent-insoluble fraction, which contained numerous ubiquitinated proteins, and in which the level of G64D-V5 was greater than that of WT-V5. These findings indicated that ZIP13 is normally degraded by a proteasome-dependent pathway, and that the G64D mutation alters the protein's properties so that more of it accumulates in the detergent-insoluble fraction (Fig 3A, right).
- line 12 on page 6
"In addition, we noted an increase in the slowly migrating ubiquitinated wild-type ZIP13 protein after MG132 treatment (Fig 4B, left), and that the ubiquitinated/non-ubiquitinated G64D protein ratio was significantly higher than that of wild type (Fig 4B, right)."
- line 15 on page 7
"In addition, a VCP inhibitor DBeQ (Chou et al, 2011) could suppress the decay of the $ZIP13^{G64D}$ protein (Fig 6F). These findings suggested that the VCP-linked proteosome-dependent pathway is involved in the normal steady-state turnover of wild-type ZIP13, and is critical for the clearance of the pathogenic mutant ZIP13 protein."

Q9). The deltaFLA appears more unstable and its levels return to those of wt (fig 5f). Since the authors have ruled out transcription I am wondering if there is also some translational control.

A9: We thank the reviewer for this important comment. We added the following sentence to show that we have not excluded the possibility of defects in translation and/or additive degradation pathway(s).
- line 9 on page 9
"Moreover, we cannot exclude the possible involvement of another degradation pathway or translational defects of the ZIP13 mutants as a consequence of the mutation, given that the $ZIP13^{\Delta FLA}$ protein level recovered much more than the $ZIP13^{G64D}$ protein level after MG132

treatment (Fig 5F and H) although the ZIP13$^{\Delta FLA}$ protein was more unstable than the ZIP13^{G64D} protein (Fig 5G)."

Q10). Pg 7. "...indicating that the VCP protein might preferentially interact with pathogenic ZIP13G64D."
Q11). Pg 8. The argument that VCP may recognize a conformational change caused by the mutation seems unlikely since the wild type is also ubiquitinated and also appears to be degraded by the proteasome.

A10-11: We appreciate these important points, which are related to Q8. We amended the sentences as suggested, as follows.
- line 18 on page 8
"VCP associates with either wild-type or mutant ZIP13 proteins, even though it preferentially interacts with the mutant ZIP13, suggesting that the VCP-ZIP13 interaction is important for both the normal steady-state turnover of wild-type ZIP13 and the clearance of ZIP13 proteins containing critical mutations (Fig 6)."

Q12). The bortezomib results are a little weak, particularly with the deltaFLA mutant (fig 7AB). Why is this?

A12: Thank you for this important comment, which may be related to Q9. The previous data may not have been convincing due to some technical matters. We now have clearer data, which are presented in the new Fig E8. We do not exclude the possibility that each 26S proteasome inhibitor might have unique and/or additional pharmacological mechanisms. We also cannot rule out the possibilities of translation defects and/or the use of additional degradation pathway(s) by the DFLA mutation, as discussed above, so we added the following sentence in line 39 on page 6
"26S proteasome inhibitors could restore the impaired intracellular Zn homeostasis of the ZIP13 mutants; thus, the manipulation of 26S proteasome activity by inhibitory compounds might be a therapeutic approach for SCD-EDS caused by pathogenic mutant ZIP13 proteins."

Q13). I do not find the model (fig 8) very convincing given the data. Why do the authors think the mutants insert in the membrane (at which point they would seem to be stabilized and/or difficult to direct the proteasome) rather than improperly folding (e.g. such that the typically hidden VCP binding domain is revealed) and hence targeted for the proteasome before reaching the membrane?

A13: We appreciate this reviewer's suggestion. To avoid confusion regarding our conclusion, we simplified to present this issue as a chart in the new Fig 7.

Again, I thank for this reviewer's highly constructive comments.

Reviewer #2:
We thank Reviewer #2 for her/his valuable comments and suggestions for our work, particularly regarding its impact on zinc transporter and physio-pathogenesis, by mainly pointing out the need for quantification of the intracellular zinc level, and another cell model that reflects SCD-EDS. These points helped us to improve our manuscript considerably.

Q1). P4, L5, the metallothionein 1 gene as an indicator for the cytosolic Zn level is indirect. More direct evidence should be included such as AAS, ICPMS, or fluorescence detection.
Q2). P4, L9, the authors should show the effect of ZIP13G64D on Zn levels, which directly reflects the Zn homeostasis.

A1-2: We thank the reviewer for these suggestions. We measured the intracellular zinc level by Inductively Coupled Plasma-Atomic Emission Spectrometry (ICP-AES) and by fluorescence detection using flow cytometry. These results are shown in the new Figs E1 and E9, respectively.

Q3). SCD-EDS mainly involved connective tissues; therefore validation on cell lines derived from connective tissue may be more relevant to further clarify the relationship between ZIP13 mutations and the disease.

A3: We thank the reviewer for this important comment, which is similar to a comment from Reviewer #1. We performed additional experiments using a human dermal fibroblast and the human fibroblast cell line HT1080. We also obtained a result that was consistent with those obtained using 293T and HeLa cells. This result is shown in the new Fig 5H and E3.

Q4). Fig. 4C, the figure legend and the right panel is inconsistent (including DbeQ or not), statistics is also strongly suggested for this figure.

A4: We appreciate the comment. We corrected the sentences as suggested.

Q5). The result showed that the proteasome inhibitor can increase the expression level of $ZIP13^{G64D}$ and $ZIP13^{\Delta FLA}$*, the two mutant proteins. The author should clarify whether these mutant proteins restore their normal zinc uptake function.*

A5: This is an important question that we would truly like to address, related to question 2 (Q2). Fluorescence detection using confocal microscopy and flow cytometry, and *MT1* promoter reporter assay revealed that the proteasome inhibitor treatments increased the intracellular zinc level in each mutant expressing cell (Figs E8 and E9). In addition, actually we had tried to purify and isolate the mutants as recombinant proteins from baculo-expression systems that we have already established (Bin et al. J Biol Chem. 2011, 286: 40255-40265.), to assess their transport ability directly using proteoliposome techniques. Unfortunately, however, most of the mutant proteins form aggregates, so we could not obtain enough soluble protein to perform these analyses. Nevertheless, because this is such an important issue, we have discussed it in the revised manuscript as follows. Again, thank you very much for this comment.
- line 14 on page 9
"In addition, the development of a direct Zn transport assay system using proteoliposomes with purified mutant ZIP13 proteins may also facilitate elucidation of the physio-pathogenesis of ZIP13."

Q6). P5, L20, IRES need to be spelled out. IRES (internal ribosome entry site)

A6: Thank you for the comment. We spelled it out as suggested.

Q7). Several important studies were missing in the reference list on zinc transporters (ZIP13, ZIP4, ZIP6, etc.) in various diseases. i.e. Potocki J Inorg Biochem 2013, Ou J Cell Biochem 2012, Zhang EMBO Mol Med 2013, Li PNAS 2007, Taylor Mol Med 2007, Hogstrand Biochem J 2013, Aydemir Gastroenterology 2012, Ryu PNAS 2011. Ref.

A7: We appreciate this informative suggestion. We cited all the suggested papers, as well as the following papers on ZIP8 and osteoarthritis (Kim JH et al, Cell 2014) and ZIP4's role in intestinal integrity (Geiser J et al, PLoS Genet 2012), instead of Ou J Cell Biochem 2012, since unfortunately we could not find this paper.

Q8). The manuscript is lengthy, suggest shortening it especially the results section.

A8: We thank you for this important comment. We have shortened the manuscript throughout the sections, and hired a professional English editing service to help make the writing more concise.

I am very grateful for this reviewer's highly constructive comments.

Referee #3

I deeply appreciate Reviewer #3's supportive comments, in particular regarding the relevance of zinc transporter-mediated zinc homeostasis from the clinical and pharmacological viewpoints. This

reviewer mainly commented about the relevance of the ubiquitination and VCP association of wild-type ZIP13 in the protein's normal homeostasis, about the need for a quantitative analysis of the intracellular zinc level, and suggested we tone down the statements about the clinical potential of proteosome inhibitors.

Q1). The only pieces of data that appear contradictory to the authors thesis are the obvious ubiquitylation of wild-type ZIP13 and its association with VCP: figure 4B and figures 6A-6B, respectively. However, this apparent discrepancy can be readily explained by normal steady-state turnover of wild-type ZIP13, presumably occurring through the same/similar pathway. In addition, there is obviously more wild-type protein in these experiments, contributing to the appearance of increased ubiquitylation and VCP association to wild-type ZIP13. I would recommend that the authors address these data more thoroughly in their manuscript so that readers are not unnecessarily confused by the findings.

A1: We thank the reviewer for this important comment, which is related to questions from Reviewer #1. As shown in Figure 3A and G, the G64D mutation leads to its accumulation in the detergent-insoluble fraction, most likely due to changes in the properties of the ZIP13 protein. To avoid confusion, we now show the ubiquitinated/non-ubiquitinated protein ratio in the new Figure 4B and Fig E6, which demonstrate that more ubiquitination occurs on the mutant proteins than on the wild-type ZIP13, supporting the idea that the G64D protein is more unstable and easily ubiquitinated than the wild-type one. Since we greatly appreciate the suggestion from this reviewer that "*this apparent discrepancy can be readily explained by normal steady-state turnover of wild-type ZIP13, presumably occurring through the same/similar pathway*", we have added the following sentences, which we believe to clarify this issue.

- line 14 on page 5
"While Bafilomycin had no apparent effect on the protein expression patterns, MG132 preferentially increased the amount of WT-V5 and G64D-V5 protein in the NP40-detergent-insoluble fraction, which contained numerous ubiquitinated proteins, and in which the level of G64D-V5 was greater than that of WT-V5. These findings indicated that ZIP13 is normally degraded by a proteasome-dependent pathway, and that the G64D mutation alters the protein's properties so that more of it accumulates in the detergent-insoluble fraction (Fig 3A, right)."

- line 12 on page 6
"In addition, we noted an increase in the slowly migrating ubiquitinated wild-type ZIP13 protein after MG132 treatment (Fig 4B, left), and that the ubiquitinated/non-ubiquitinated G64D protein ratio was significantly higher than that of wild type (Fig 4B, right)."

- line 10 and page 7
"Intriguingly, the ratio of VCP associated with $ZIP13^{G64D}$ was higher than that of VCP associated with $ZIP13^{WT}$ (Fig 6B, lower), indicating that the VCP protein might preferentially interact with the pathogenic $ZIP13^{G64D}$ protein."

- line 15 on page 7
"In addition, a VCP inhibitor DBeQ (Chou et al, 2011) could suppress the decay of the $ZIP13^{G64D}$ protein (Fig 6F). These findings suggested that the VCP-linked proteosome-dependent pathway is involved in the normal steady-state turnover of wild-type ZIP13, and is critical for the clearance of the pathogenic mutant ZIP13 protein."

- line 9 on page 9
"Moreover, we cannot exclude the possible involvement of another degradation pathway or translational defects of the ZIP13 mutants as a consequence of the mutation, given that the $ZIP13^{\Delta FLA}$ protein was more unstable than the $ZIP13^{G64D}$ protein (Fig 5G)."

Q2). In figure 7 (more below), and in the discussion, the authors place a significant degree of attention upon the potential role of proteasome inhibitors, such as Velcade, as potential drug candidates to alleviate some of the symptoms of this Ehlers Danlos Syndrome. However, therapeutic interventions of diseases with similar molecular pathogenesis (e.g. cystic fibrosis), have not taken this approach (likely due to the severe side effects associated with prolonged proteasome inhibitor treatment in humans, such as peripheral neuropathy). Instead, molecular "potentiators" and

"correctors" are in the clinic or in development (these drugs/drug candidates either allow the small fraction of mutant CFTR to function more effectively at the cell surface, or allow more mutant CFTR to reach the cell surface, respectively; an example potentiator is Kalydeco, for treatment of patients with the G551D CFTR mutation). The authors cite VX-809, which is in the "corrector" class. Given the obvious parallels of this study to CFTR mutations, I think it would be informative to the reader if the authors included additional detail in their discussion section to cover the therapeutic strategies found to be successful in treating cystic fibrosis, as well as some of the still experimental strategies (it is noted that VX-809 is included in their discussion).

A2: We thank the reviewer for these insightful suggestions, which are valuable for improving our detailed discussion about cystic fibrosis and CFTR, as follows.
- line 35 on page 8
"Cystic fibrosis is a genetic disease caused by mutations in the cystic fibrosis transmembrane conductance regulator (CFTR). Ninety percent of the patients have a ΔF508 mutation, which prevents proper folding and processing of the CFTR protein; as a result, little of the mutant protein reaches the cell surface (Riordan et al, 1989; Rommens et al, 1988; Ward et al, 1995). Much research has focused on elucidating the folding, trafficking, and degradation properties of CFTR pathogenic mutants, and on developing drugs that are either "potentiators" of CFTR itself or "correctors" of its degradation pathway (Becq, 2010; Gee et al, 2011; Wang et al, 2008). VX-809 is the latest CFTR drug. It was obtained from a screen as a compound that reduces degradation of the ΔF508 mutant protein and increases CFTR accumulation on the cell surface, and is currently in clinical trials (Van Goor et al, 2011). Another mutation, G551D, which accounts for about 5% of the cystic fibrosis patients, does not affect the protein's trafficking, but prohibits proper channel gating. Kalydeco (VX-770) was developed to treat cystic fibrosis patients carrying the G551D mutation (Accurso et al, 2010; Van Goor et al, 2009). It acts as a "potentiator" to open the gate of CFTR for proper chloride transport (Rowe & Verkman, 2013). In the case of SCD-EDS patients, therapeutic strategies analogous to those used to treat cystic fibrosis, as either molecular "potentiators" or "correctors," may be effective depending on the functional consequences of the mutation. Moreover, we cannot exclude the possible involvement of another degradation pathway or translational defects of the ZIP13 mutants as a consequence of the mutation, given that the $ZIP13^{\Delta FLA}$ protein level recovered much more than the $ZIP13^{G64D}$ protein level after MG132 treatment (Fig 5F) although the $ZIP13^{\Delta FLA}$ protein was more unstable than the $ZIP13^{G64D}$ protein (Fig 5G). Future investigations of the detailed mechanisms underlying the degradation of G64D and ΔFLA mutants, and of the structure of ZIP13 will improve our ability to develop therapies for SCD-EDS. In this regard, mutant *ZIP13* gene knock-in mouse models could be useful for developing treatments for SCD-EDS. In addition, the development of a direct Zn transport assay system using proteoliposomes with purified mutant ZIP13 proteins may also facilitate elucidation of the physio-pathogenesis of ZIP13."

Q3). The major problem with the data in figure 7c is that they are not quantitative. I would recommend that the authors consider identifying a quantitative approach to determining intracellular zinc levels that would complement the microscopy experiments, or simply remove figure 7c from the manuscript (As mentioned above, it is this reviewer's opinion that the potential is small for current proteasome inhibitors like Velcade to be viable therapeutic candidates for treatment of diseases like EDS). Figures 7A and 7B make the point that Velcade works similarly to research use only proteasome inhibitors.

A3: We thank the reviewer for these important comments. In fact, Bortezomib works similarly to proteasome inhibitors that are restricted to research use. After careful consideration of the reviewer's comments, we performed the intracellular zinc quantification (Figs E1 and E9) and moved Figure 7C to Expanded view Figure 8. We also discussed potential treatment strategies for SCD-EDS in the context of those used for CFTR, from line 7 on page 9.

Q4). Regarding the quality of the article's English language grammar, I would recommend that the authors have the manuscript further edited by an English language expert.

A4: We appreciate this reviewer's advice. We had the manuscript further edited by a professional English language editing service.

Again, I thank for this reviewer's highly constructive comments, especially valuable advices from clinical and pharmacological viewpoints.

5th Editorial Decision 12 May 2014

Thank you for the submission of your revised manuscript to EMBO Molecular Medicine. We have now received the enclosed reports from the referees that were asked to re-assess it. The reviewers are now supportive and I am pleased to inform you that we will be able to accept your manuscript pending final important editorial amendments:

1) Please provide figures of sufficient quality to avoid delays further on during production. As noted earlier during the review process, the resolution and quality of a number of images remains rather low. Specifically:

Fig. 1, Panel C: The resolution is too low (bands too blocky/blurry)
Fig. 2, Panel E: "Input" blot, contrast setting too high; Panels F and G, resolution too low (bands too blocky/blurry)
Fig. 3, Panels F, G and H: Resolution too low (bands too blocky/blurry)
Fig. 4, Panel A: Contrast setting too high and resolution too low (bands too blocky/blurry); Panel B "tubulin" blots, contrast setting too high
Fig. 5, Panel H: Resolution too low (bands too blocky/blurry)
Fig. 6, Panels, B. D, E and F: Resolution too low (bands too blocky/blurry)

Naturally, it is hard for us say what the actual issue is here without more information on how these images were generated (perhaps a bad JPEG to TIFF conversion?), but in the worst case you may need to reacquire the images and recompose the figures. If unresolved, these issues will lead to problems when the production team tries to resize these images for the final manuscript. You may refer to our guidelines for Authors at embomolmed.org.

2) As per our Author Guidelines "Since for complex biological experiments the number of independent repeats of a measurement often has to be limited for practical reasons, statistical measures with a very small n are commonplace. However, statistical measures applied to too small a sample size are not significant and they can suggest a false level of significance. We recommend that the actual individual data from each experiment should be plotted if $n < 5$, alongside an error bar. In cases where n is small, a justification for the use of the statistical test employed has to be provided. Presenting a single 'typical result' of n experiments is sometimes unavoidable, but should be accompanied by an indication of the variability of data between independent experiments. If n is not based on independent experiments (that is, n merely represents replicates of a measurement), statistics may still be useful, but a detailed description of the repeated measurement is required". We note that a number of graphs (e.g. from panels 4A, C, D, 5G and 6F) do not conform to these requirements in that the number of replicates, description of variability, error measurements, etc. are missing. Please amend text, figures and figure legends where appropriate to reflect these requirements. It would be useful to provide replicate data in these instances (please consider point 3 below).

3) We are now encouraging the publication of source data, particularly for electrophoretic gels and blots, with the aim of making primary data more accessible and transparent to the reader. Would you be willing to provide a PDF file per figure that contains the original, un-cropped and unprocessed scans of all or at least the key gels used in the manuscript? The PDF files should be labeled with the appropriate figure/panel number, and should have molecular weight markers; further annotation may be useful but is not essential. The PDF files will be published online with the article as supplementary "Source Data" files. If you have any questions regarding this just contact me.

4) Every published paper now includes a 'Synopsis' to further enhance discoverability and are displayed on the journal webpage. They include a short standfirst - to be written by the editor - as well as 2-5 one-sentence bullet points that summarise the paper (to be written by the author). Please provide the short list of bullet points that summarise the key NEW findings. The bullet points should be designed to be complementary to the abstract - i.e. not repeat the same text. We encourage

inclusion of key acronyms and quantitative information. Please use the passive voice. Please attach these in a separate file or send them by email, not in the manuscript, we will incorporate them accordingly.

Please submit your revised manuscript within two weeks.

***** Reviewer's comments *****

Referee #1 (Remarks):

The authors have adequately addressed my concerns, resulting in an improved manuscript that I now find suitable for publication in EMBO Molecular Medicine. The conclusions are supported by the results and address a medically relevant topic that is likely to be of interest to a wide audience.

Referee #2 (Remarks):

The authors have adequately addressed all the concerns. No more questions.

Referee #3 (Comments on Novelty/Model System):

Technical quality: The authors improved the technical quality of their manuscript by addressing reviewer concerns.

Novelty: As mentioned for the original manuscript, the fact that ZIP13 proteins are degraded via VCP is not unexpected; however this is the first time it has been demonstrated using disease-relevant mutations.

Medical impact: Medical researchers can use this information,combined with experience from CF field, to device strategies to employ "potentiators" and/or "correctors" to treat diseases with this etiology.

Adequacy of the model system: the authors addressed reviewer concerns, adding more relevant human cell lines.

Referee #3 (Remarks):

In this reviewers opinion, the authors of the manuscript entitled, "Molecular pathogenic basis of Spondylocheirodysplastic Ehlers-Danlos syndrome caused by mutant ZIP13 proteins" by Bin et al. have revised their manuscript to sufficiently address reviewer concerns. Namely, the authors addressed the following major deficiencies from their original manuscript:

1. They showed in figure 3 that mutant ZIP13 protein accumulates in an insoluble form and that, as a percentage, relatively more mutant ZIP13 protein is ubiquitylated (Figs 4B and E6).

2. They addressed reviewer concerns that about restoration of intracellular zinc levels by performing quantitative experiments (ICP and flow cytometry: figures E1 and E9). In addition their qualitative intracellular microscopy experiments detecting Zn levels are more convincing (fig E8).

3. They added the important discussion that VCP appears to be involved in the steady state turnover of WT ZIP13 as well as mutant ZIP13. As I noted before, this explanation for some of their results (stabilization of ubiquitylated WT and WT association with VCP) is entirely consistent with our understanding of WT and mutant transmembrane protein metabolism.

This reviewer has no further requests for revisions from the authors.

2nd Revision - authors' response 26 May 2014

Responses to requests:

1) *Please provide figures of sufficient quality to avoid delays further on during production. As noted earlier during the review process, the resolution and quality of a number of images remains rather low. (Fig. 1C, Fig. 2E(Input" blot), F and G, Fig. 3 F, G and H, Fig. 4A and B (tubulin blot), Fig. 5H, Fig. 6B, D, E and F)*

-A1: We have prepared the indicated images with better quality, some of which were obtained by re-performing the experiments to get better images.

2) *As per our Author Guidelines "Since for complex biological experiments the number of independent repeats of a measurement often has to be limited for practical reasons, statistical measures with a very small n are commonplace. However, statistical measures applied to too small a sample size are not significant and they can suggest a false level of significance. We recommend that the actual individual data from each experiment should be plotted if n < 5, alongside an error bar. In cases where n is small, a justification for the use of the statistical test employed has to be provided. Presenting a single 'typical result' of n experiments is sometimes unavoidable, but should be accompanied by an indication of the variability of data between independent experiments. If n is not based on independent experiments (that is, n merely represents replicates of a measurement), statistics may still be useful, but a detailed description of the repeated measurement is required". We note that a number of graphs (e.g. from panels 4A, C, D, 5G and 6F) do not conform to these requirements in that the number of replicates, description of variability, error measurements, etc. are missing. Please amend text, figures and figure legends where appropriate to reflect these requirements. It would be useful to provide replicate data in these instances (please consider point 3 below).*

-A2: We have added the following sentences in the indicated figure legends as below.

Figure 4A: Data are representative of two independent experiments.

Figure 4C: The graph is representative of four independent experiments.

Figure 4D: Data are representative of two independent experiments. Similar results were obtained in a healthy male donor and male SCD-EDS patient.

Figure 5G: Data are representative of three independent experiments.

Figure 6F: Data are representative of two independent experiments.

3) *We are now encouraging the publication of source data, particularly for electrophoretic gels and blots, with the aim of making primary data more accessible and transparent to the reader. Would you be willing to provide a PDF file per figure that contains the original, un-cropped and unprocessed scans of all or at least the key gels used in the manuscript? The PDF files should be labelled with the appropriate figure/panel number, and should have molecular weight markers; further annotation may be useful but is not essential. The PDF files will be published online with the article as supplementary "Source Data" files. If you have any questions regarding this just contact me.*

-A3: We have deposited all of the original electrophoretic gels and blots images in the main figures that are key results for our paper.

4) *Every published paper now includes a 'Synopsis' to further enhance discoverability and are displayed on the journal webpage. They include a short standfirst - to be written by the editor - as well as 2-5 one-sentence bullet points that summarise the paper (to be written by the author). Please provide the short list of bullet points that summarise the key NEW findings. The bullet points should*

be designed to be complementary to the abstract - i.e. not repeat the same text. We encourage inclusion of key acronyms and quantitative information. Please use the passive voice.

-A4: We have prepared "Synopsis" as below.

The molecular mechanisms of the Spondylocheirodysplastic form of Ehlers-Danlos syndrome (SCD-EDS, OMIM 612350) caused by mutant zinc transporter ZIP13 proteins was investigated, revealing that they are rapidly degraded by the VCP-linked ubiquitination-dependent proteasome pathway.

•Pathogenic ZIP13 mutant proteins: ZIP13^{G64D} in which Gly at amino acid position 64 is replaced by Asp, and ZIP13$^{\Delta FLA}$ which contains a deletion of Phe-Leu-Ala, are degraded by the ubiquitin-proteasome pathway.

• Valosin-containing protein (VCP) is involved in the degradation of the pathogenic mutant ZIP13 proteins.

•The reduced expression levels of the ZIP13 mutant proteins are rescued by inhibition of the degradation pathways, resulting in improved intracellular zinc homeostasis.

Minor modifications:

We added the following updated book and paper in reference citation list.

•Zinc signal in growth control and bone diseases. Fukada T, et al (2014): Springer, in press

•Histological Analysis of Dentinogenesis Imperfecta in Slc39a13/Zip13 Knockout Mice. Munemasa T, et al (2014) Journal of Hard Tissue Biology 23: 163-170

On behalf of my co-authors, I do hope that this revised manuscript is now suitable for publication in *EMBO Molecular Medicine*.

Thank you again for your attention, great help, and considerations.
