# Supplementary figures and images for: Molecular pathogenesis of Spondylocheirodysplastic Ehlers-Danlos syndrome caused by mutant ZIP13 proteins
Source: EMBO Mol Med. 2014 Jul 9;6(8):1028–42. doi: 10.15252/emmm.201303809 (PMC4154131; doi:10.15252/emmm.201303809)

Figure 1C

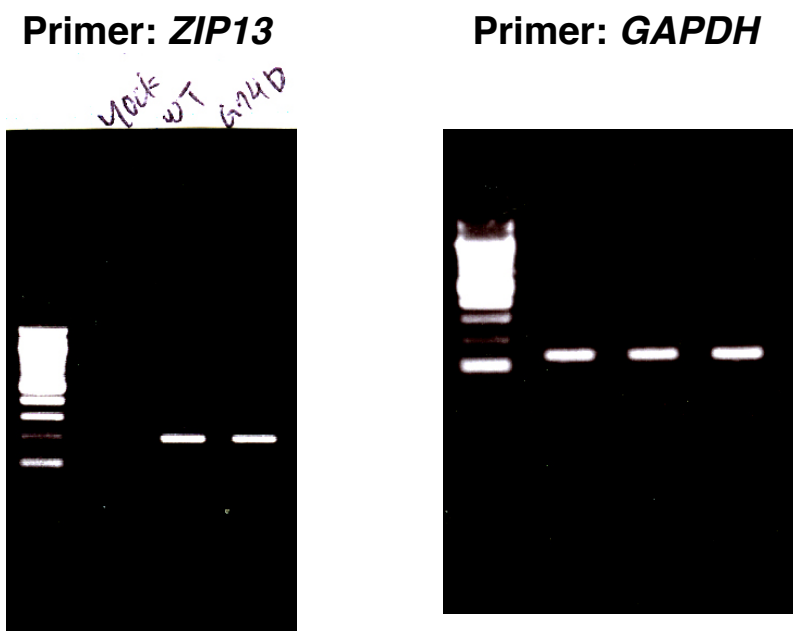

Figure 1E

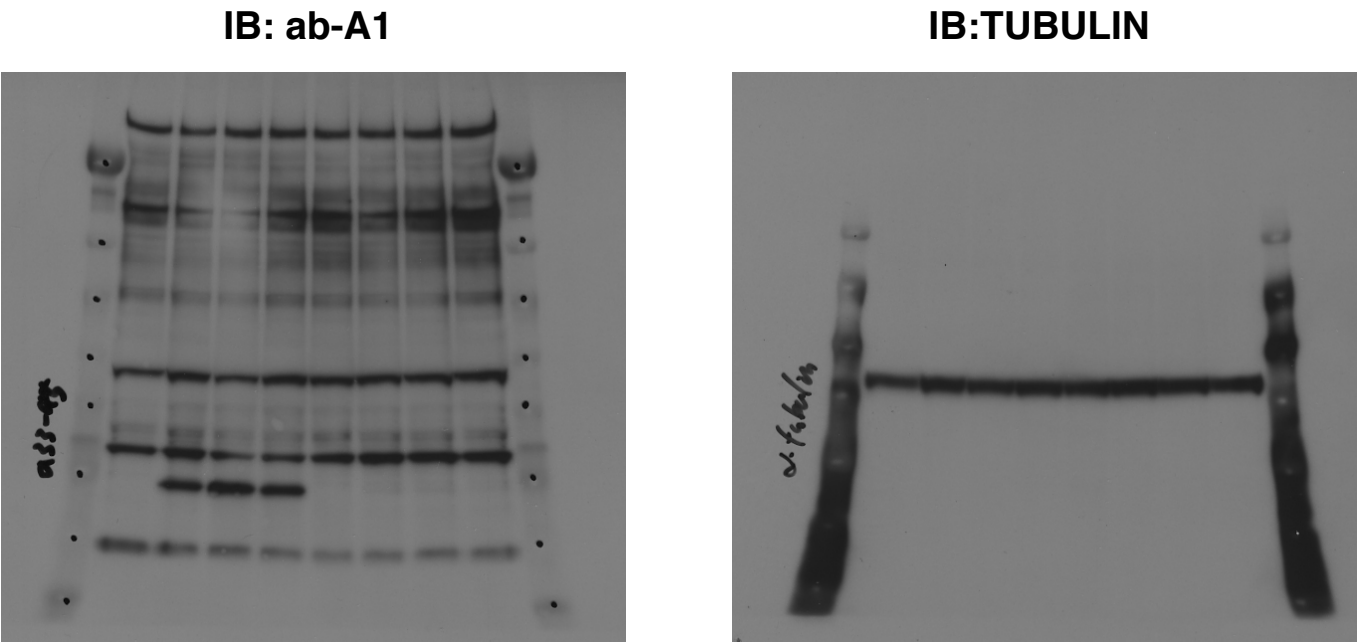

Supplement: Supplementary file 3 [file emmm0006-1028-sd3.pdf]

**Figure 2B**

**Silver staining**

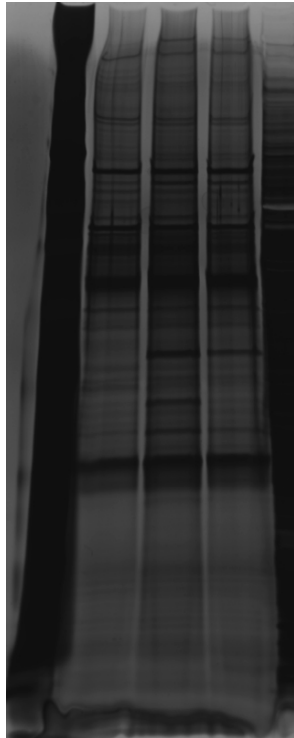

**IB: FLAG**

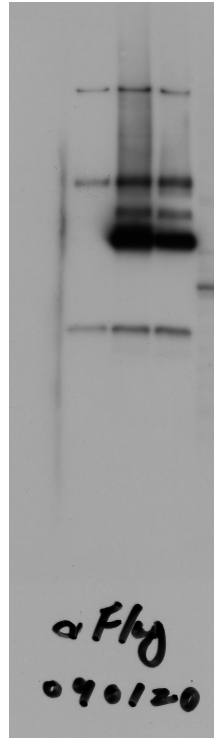

**IB: ab-A1**

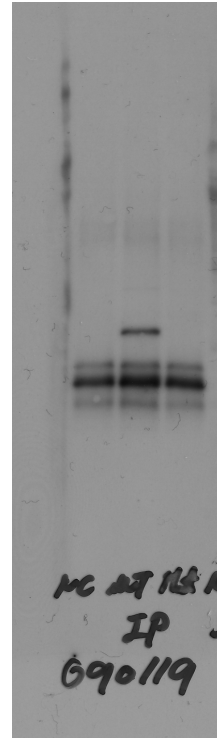

**Figure 2C**

**IB: FLAG**

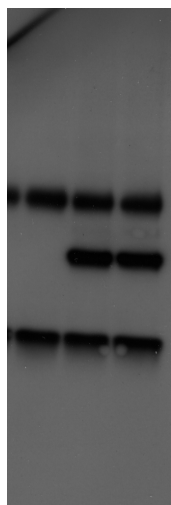

**IB: ab-A2**

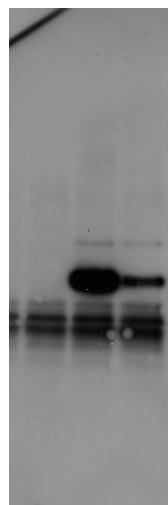

**IB: GAPDH**

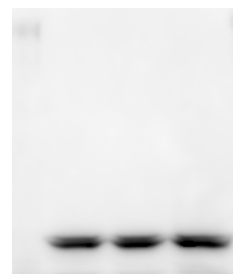

**Figure 2E**

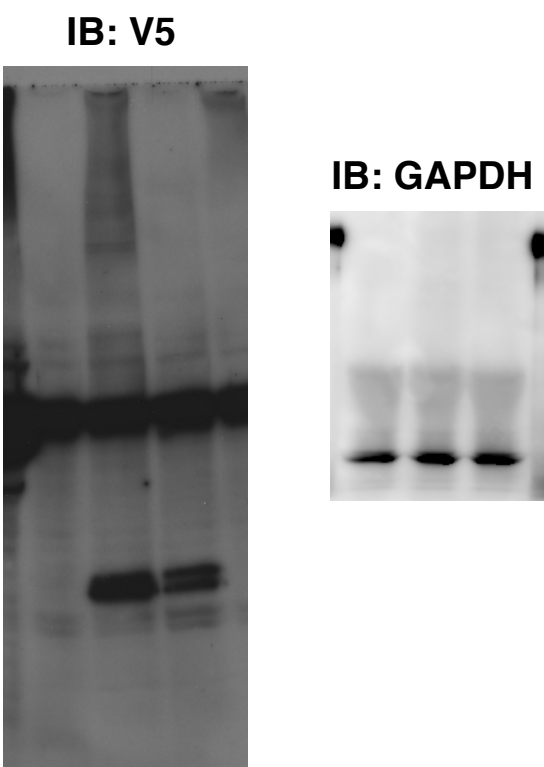

**Figure 2F**

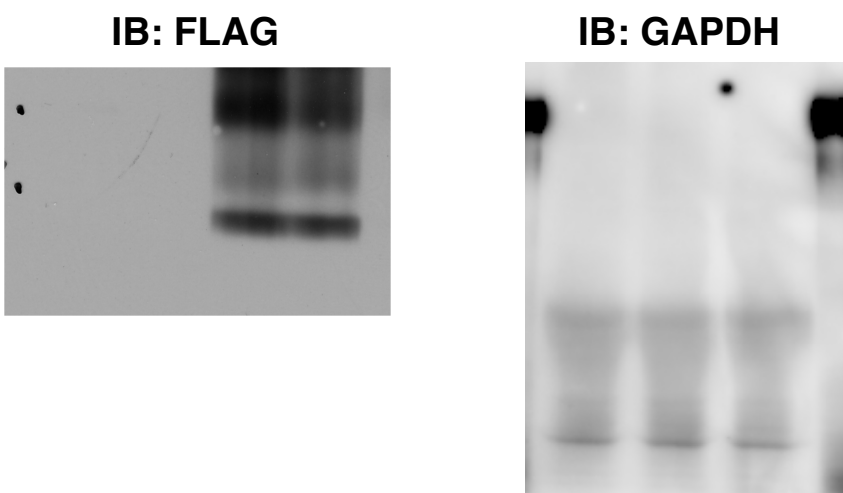

**Figure 2G**

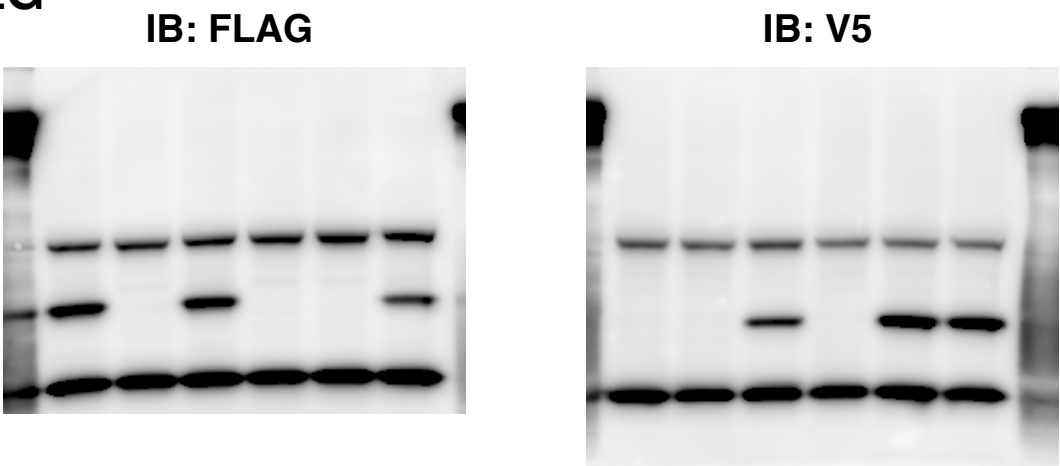

Supplement: Supplementary file 4 [file emmm0006-1028-sd4.pdf]

Figure 4A

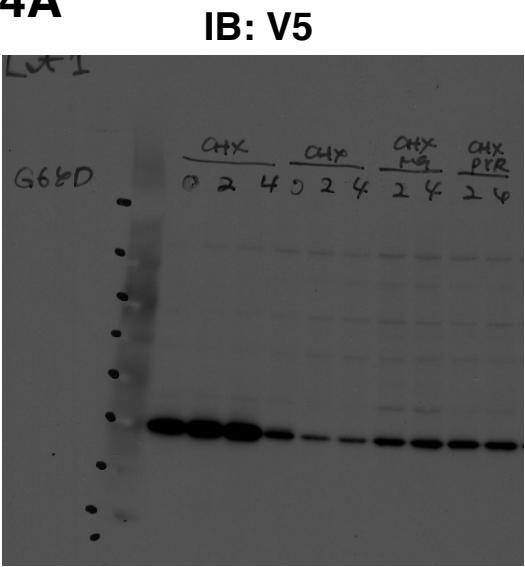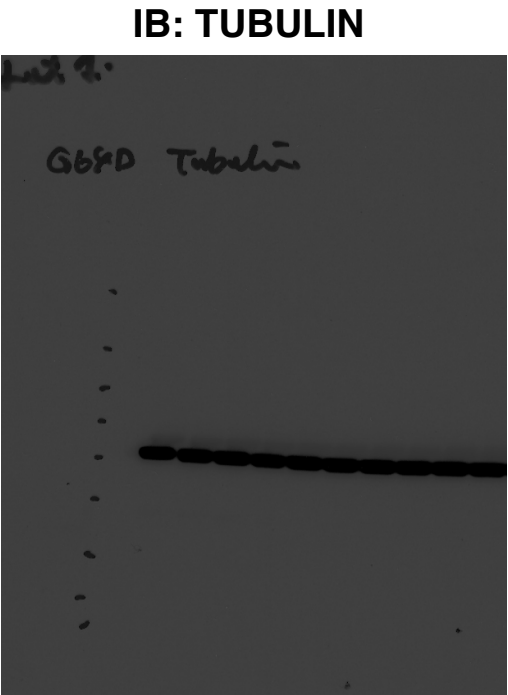

Figure 4B

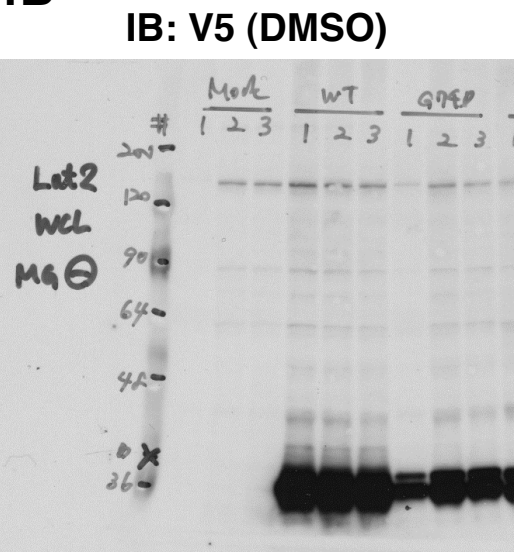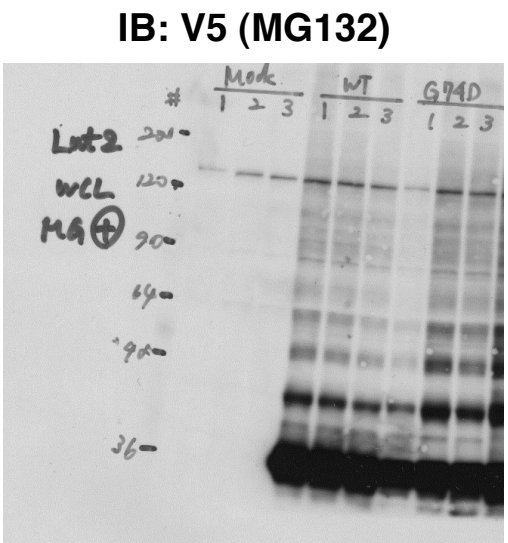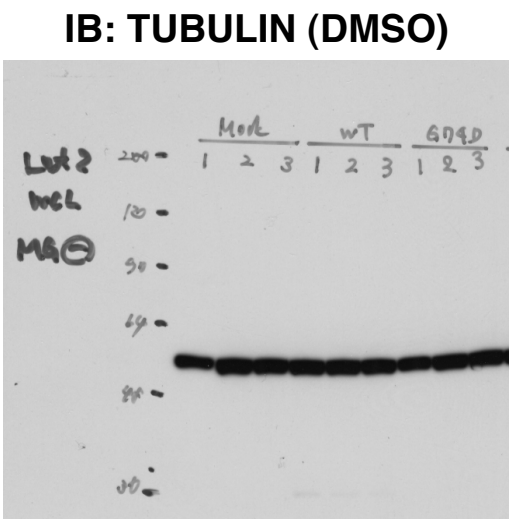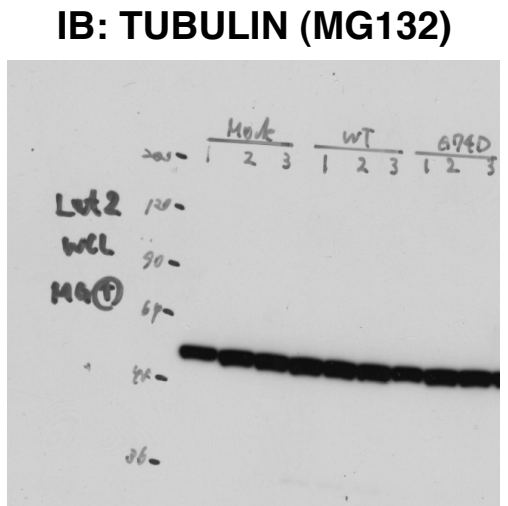

Supplement: Supplementary file 6 [file emmm0006-1028-sd6.pdf]

Figure 5C

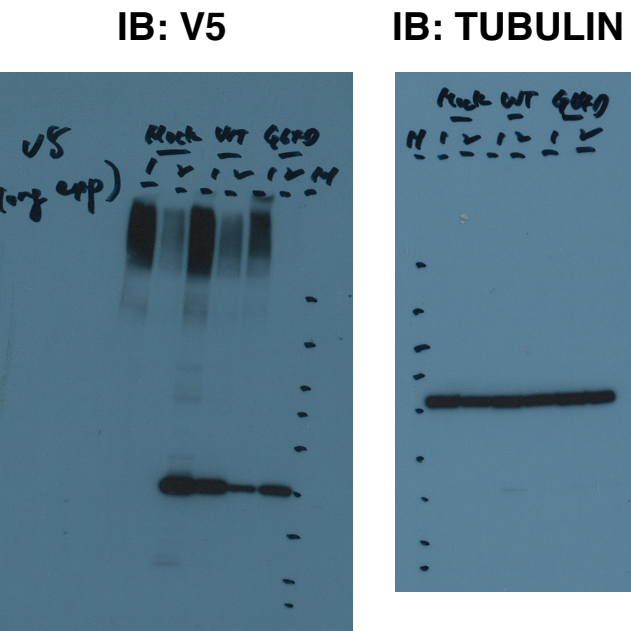

Figure 5D

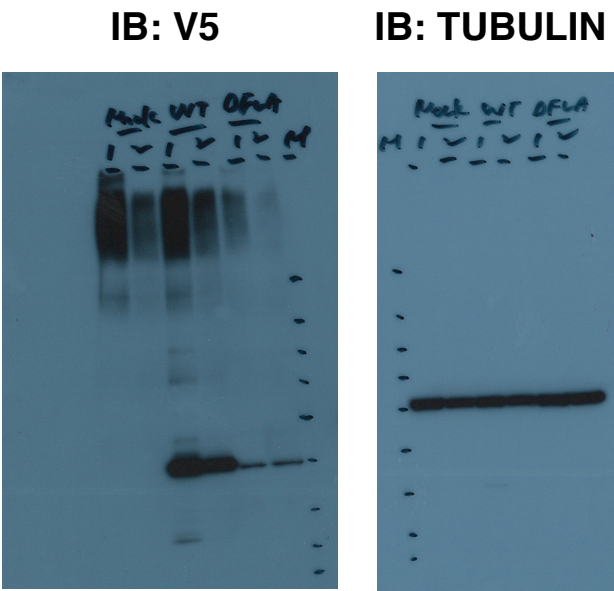

Figure 5F

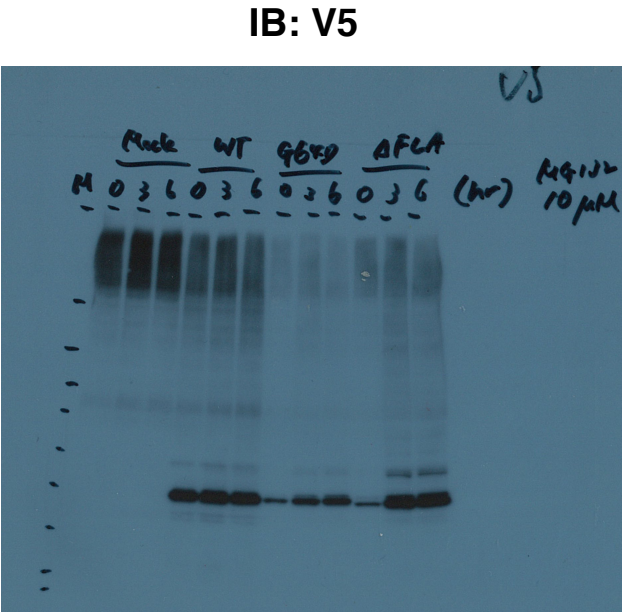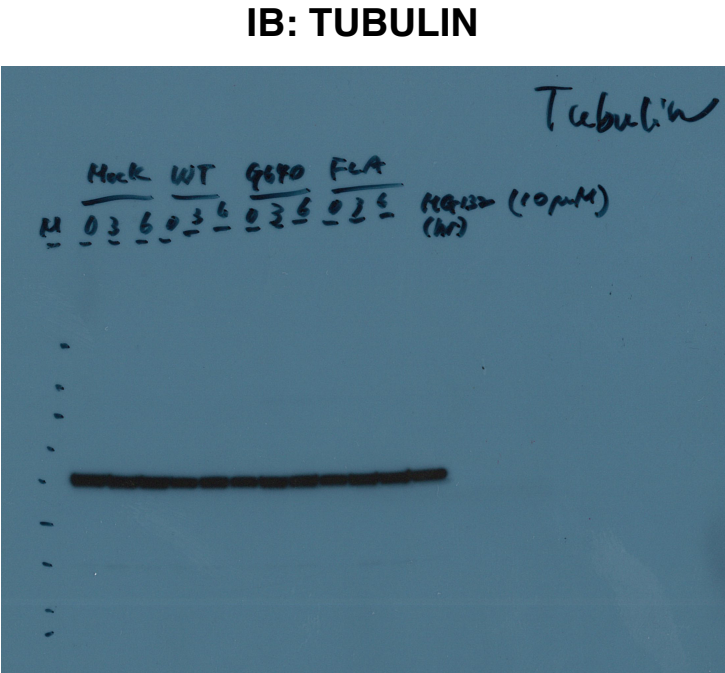

**Figure 5G**

IB: V5

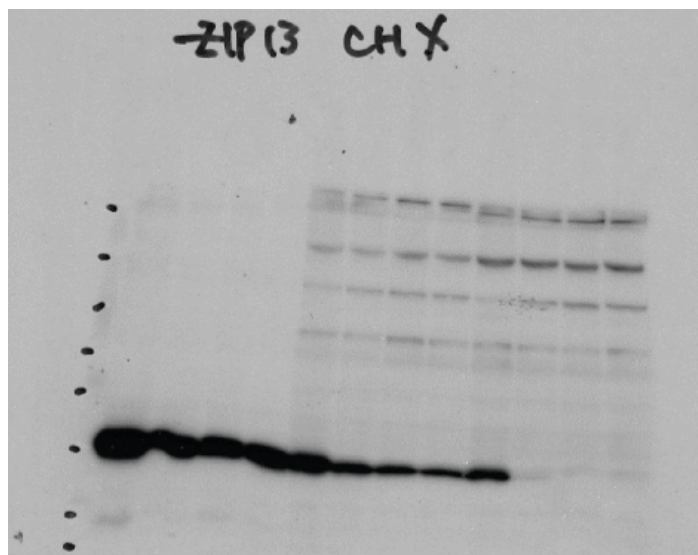

IB: TUBULIN

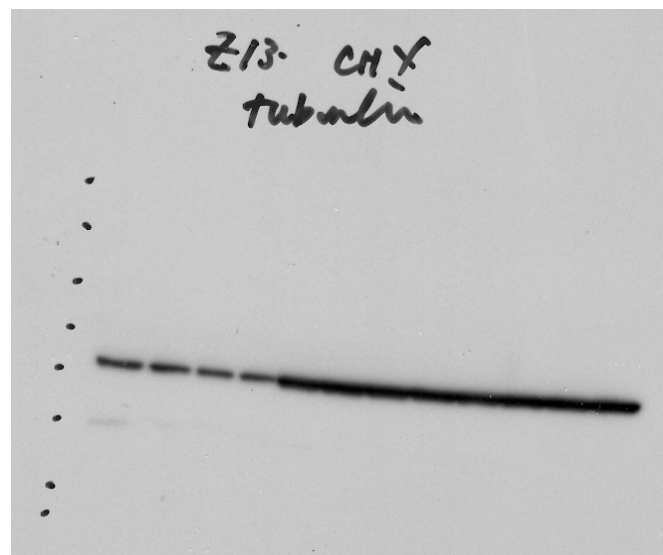

**Figure 5H**

IB: V5

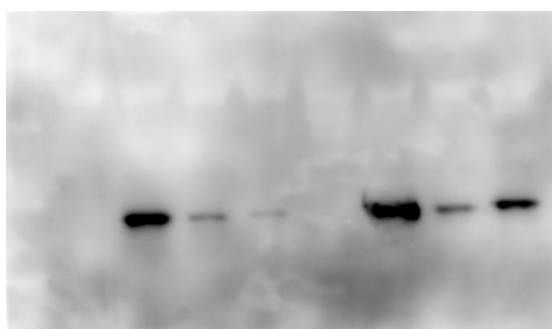

IB: TUBULIN

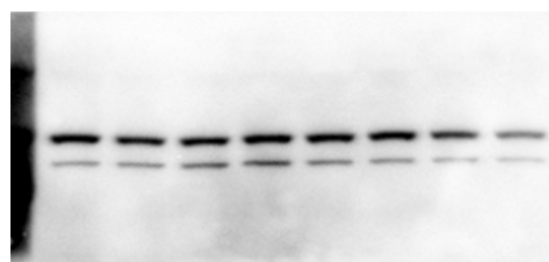

Supplement: Supplementary file 7 [file emmm0006-1028-sd7.pdf]

**Figure 6A**

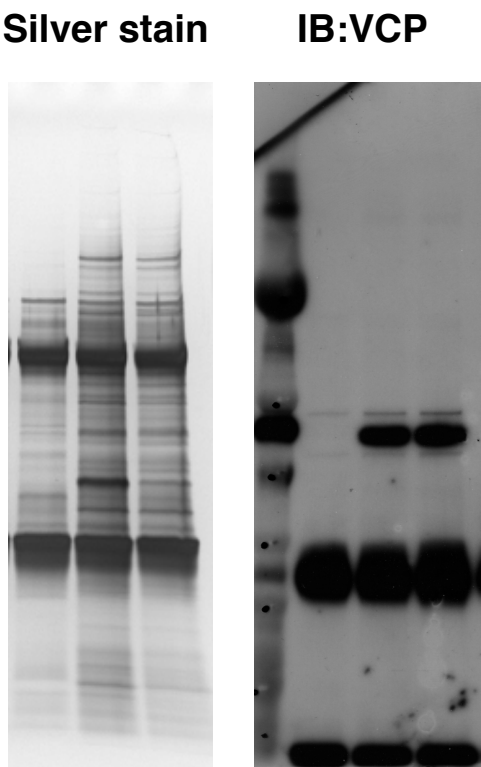

**Figure 6B**

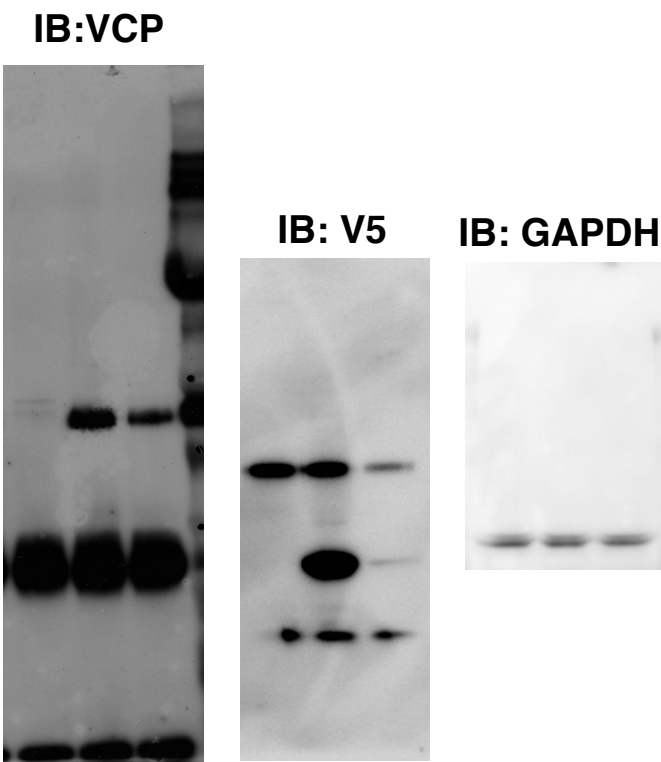

**Figure 6D**

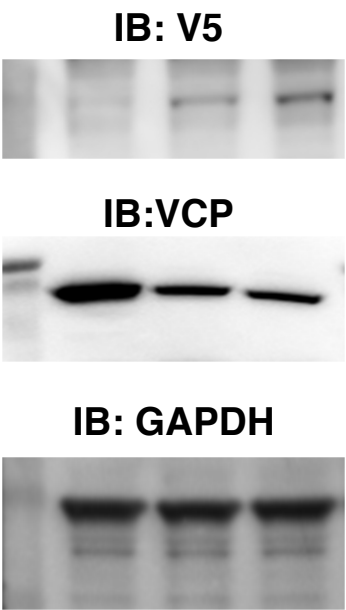

**Figure 6E**

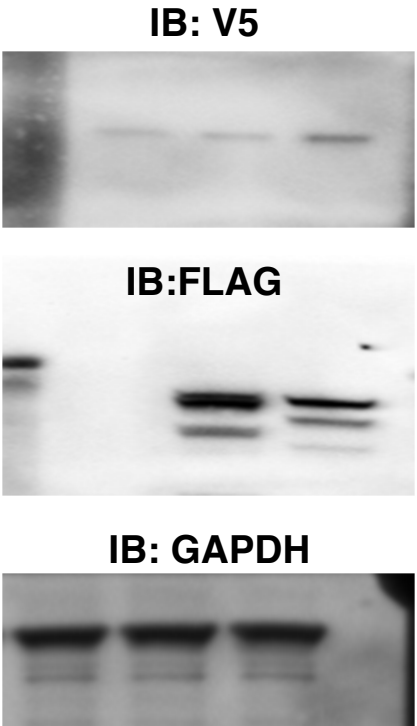

Figure 6F

IB: V5

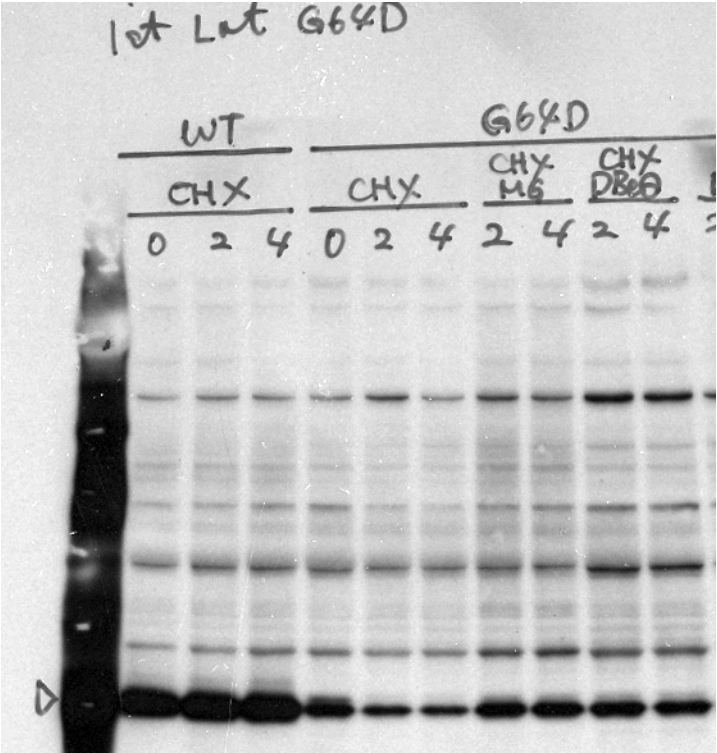

IB: TUBULIN

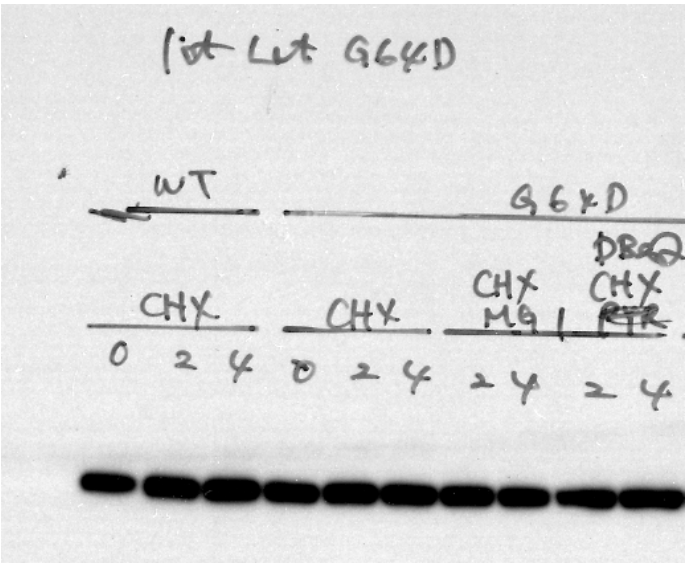

Supplement: Supplementary file 8 [file emmm0006-1028-sd8.pdf]
