# Supplementary material for: Molecular pathogenesis of Spondylocheirodysplastic Ehlers-Danlos syndrome caused by mutant ZIP13 proteins
Source: EMBO Mol Med. 2014 Jul 9;6(8):1028–42. doi: 10.15252/emmm.201303809 (PMC4154131; doi:10.15252/emmm.201303809)

Figure 3A

IB: V5 (NP40-Soluble)

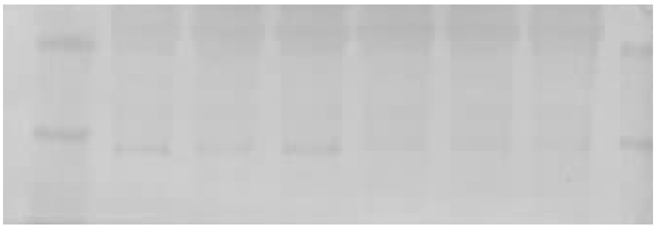

IB: Ubiquitin (NP40-Soluble)

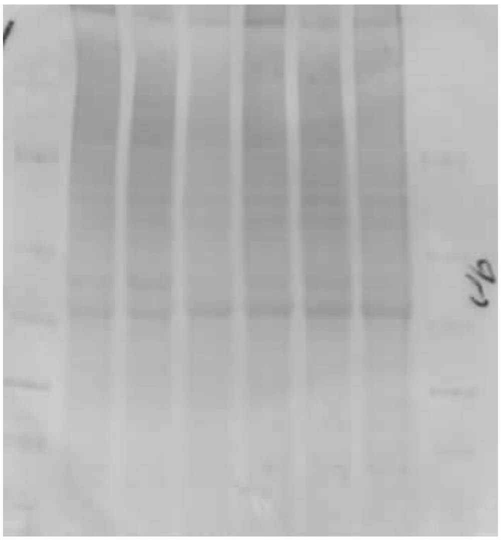

IB: GAPDH (NP40-Soluble)

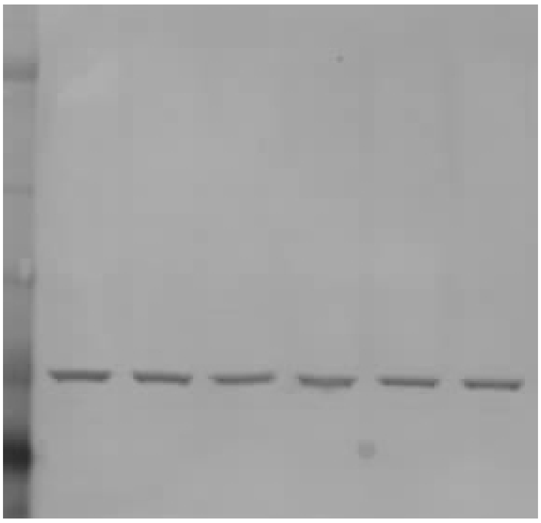

IB: V5 (NP40-Insoluble)

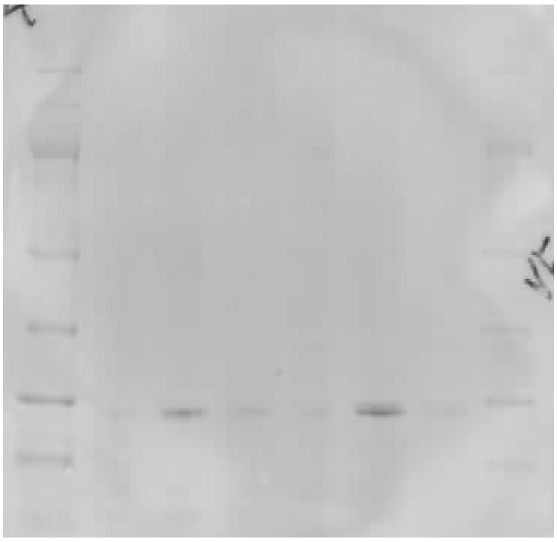

IB: Ubiquitin (NP40-Insoluble)

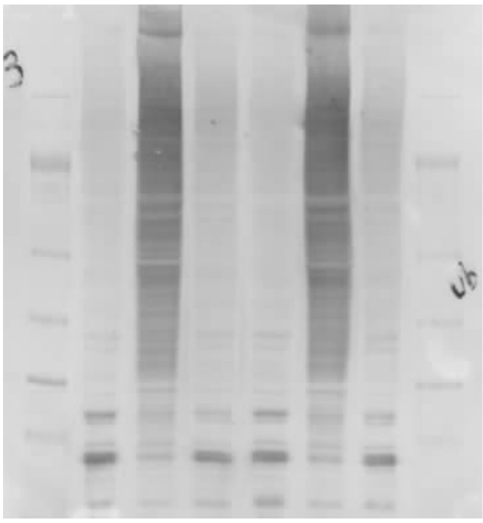

Figure 3B

IB: V5 | IB: TUBULIN

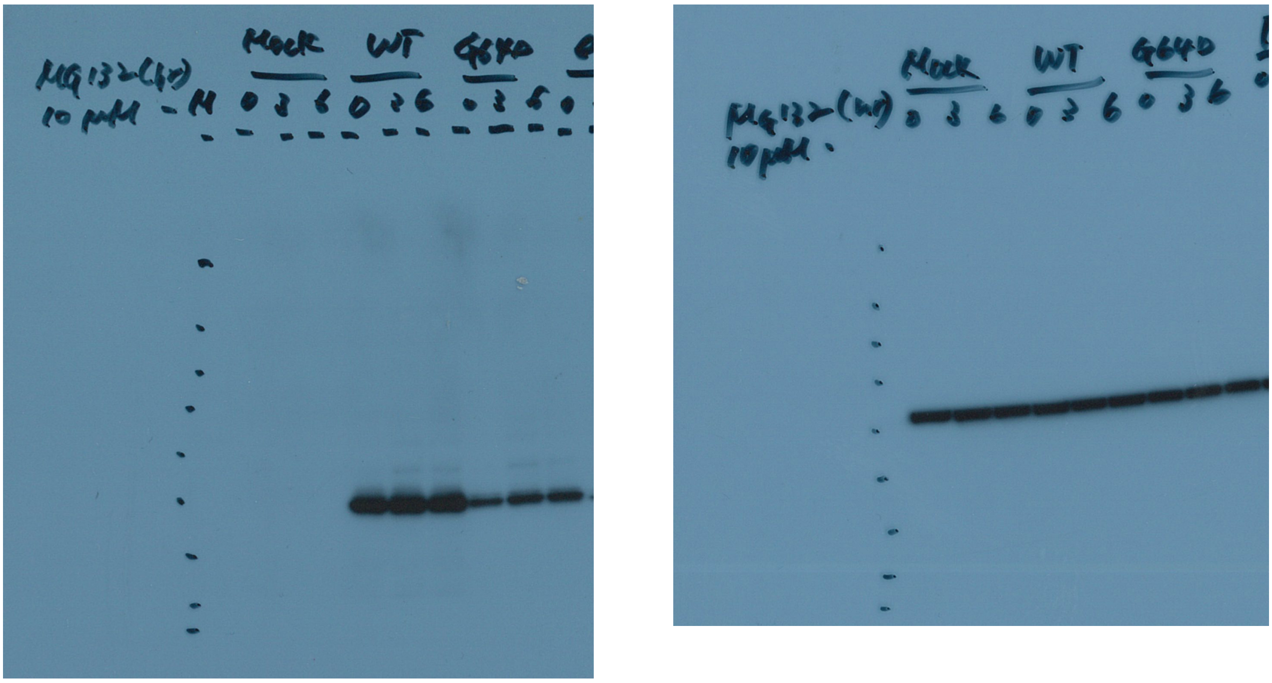

Figure 3D

IB: V5 | IB: TUBULIN

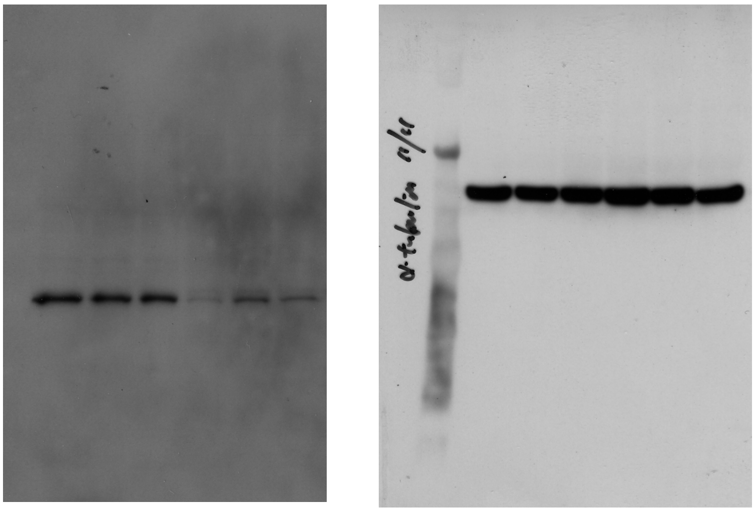

Figure 3F

IB: V5 | IB: GAPDH

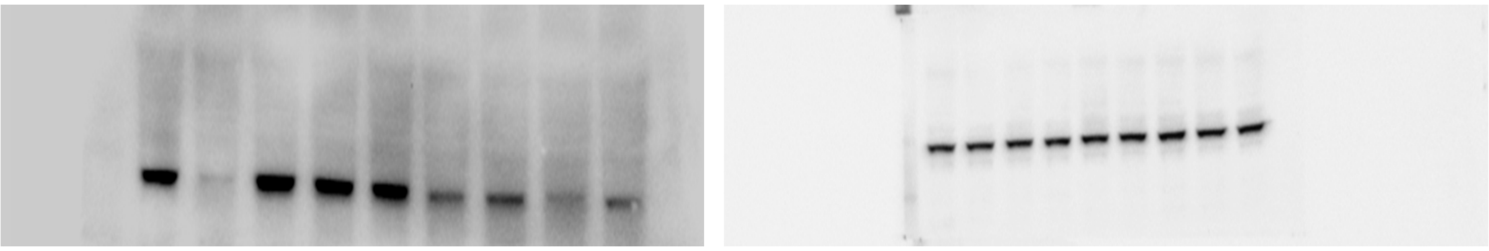

Figure 3G

IB: V5
(DMSO NP40-Soluble)

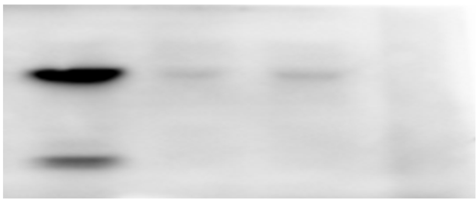

IB: V5
(MG132 NP40-Soluble)

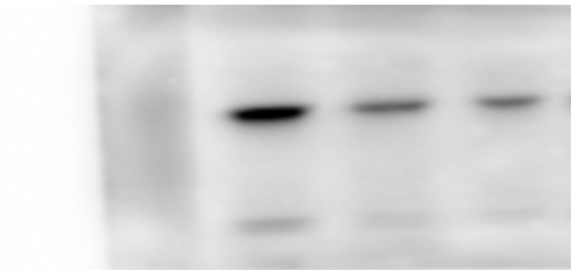

IB: GAPDH
(DMSO NP40-Soluble)

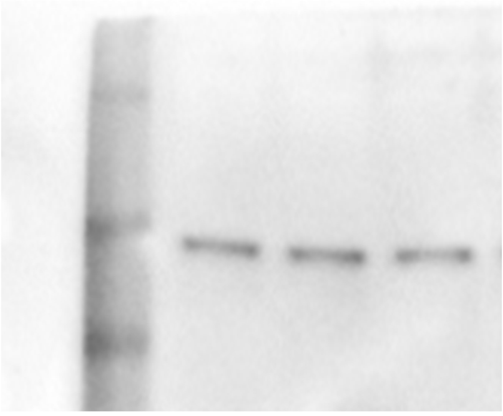

IB: GAPDH
(MG132 NP40-Soluble)

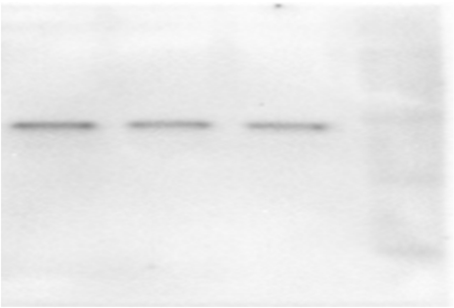

IB: V5
(DMSO NP40-Insoluble)

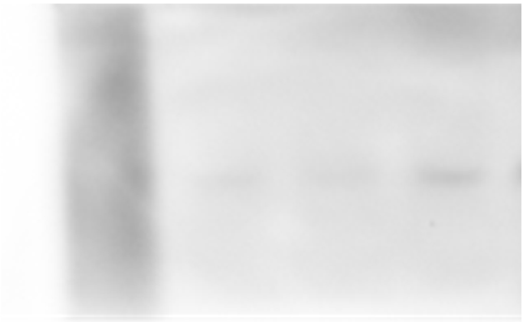

IB: V5
(MG132 NP40_Insoluble)

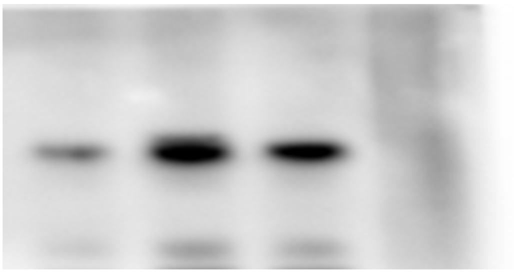

Figure 3H

IB: V5

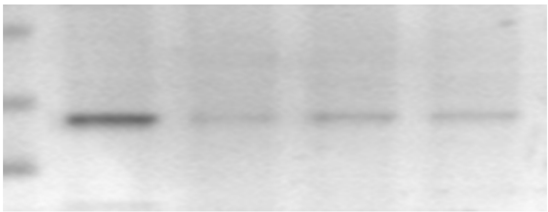

IB: GAPDH

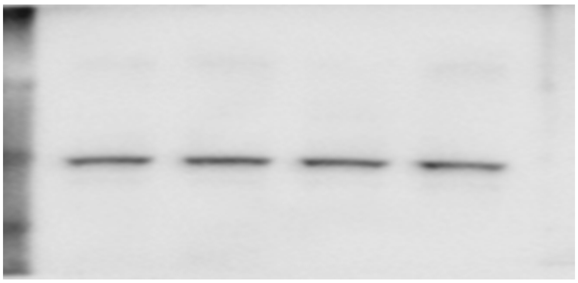

Supplement: Supplementary file 5 [file emmm0006-1028-sd5.pdf]
